# Ordinary citizens are more severe towards verbal than nonverbal hate-motivated incidents with identical consequences

**DOI:** 10.1038/s41598-023-33892-8

**Published:** 2023-05-02

**Authors:** Jimena Zapata, Ophelia Deroy

**Affiliations:** 1 Faculty of Philosophy, Philosophy of Science and Religious Studies Ludwig-Maximilian University, Munich, Germany; 2 Department of Philosophy I, University of Granada, Granada, Spain; 3 Munich Center for Neuroscience, Munich, Germany; 4 Institute of Philosophy, School of Advanced Study, University of London, London, UK

**Keywords:** Human behaviour, Social behaviour

## Abstract

**Abstract:**

Do we judge hate incidents similarly when they are performed using words or bodily actions? Hate speech incidents are rarely reported by bystanders, and whether or how much they should be punished remains a matter of legal, theoretical and social disagreement. In a pre-registered study (N = 1309), participants read about verbal and nonverbal attacks stemming from identical hateful intent, which created the same consequences for the victims. We asked them how much punishment the perpetrator should receive, how likely they would be to denounce such an incident and how much harm they judged the victim suffered. The results contradicted our pre-registered hypotheses and the predictions of dual moral theories, which hold that intention and harmful consequences are the sole psychological determinants of punishment. Instead, participants consistently rated verbal hate attacks as more deserving of punishment, denunciation and being more harmful to the victim than nonverbal attacks. This difference is explained by the concept of action aversion, suggesting that lay observers have different intrinsic associations with interactions involving words compared to bodily actions, regardless of consequences. This explanation has implications for social psychology, moral theories, and legislative efforts to sanction hate speech, which are considered.

**Protocol registration:**

The Stage 1 protocol for this Registered Report was accepted in principle on 29/06/2022. The protocol, as accepted by the journal, can be found at: 10.17605/OSF.IO/Z86TV.

## Introduction

Should we prosecute people for their words and not just their deeds? A robust liberal tradition inspired by John Stuart Mill's theories argues that Freedom of Speech, as a constitutional right, is granted with superior protection against State regulatory interference, despite possible conflicts with morality^1^. However, most European countries have introduced hate speech regulations to reframe that privilege, sanctioning verbal and nonverbal hate attacks similarly when they share analogous severity and degrading intention and create comparable consequences^2–4^. Legal theorists are not the only ones to disagree on the extent to which speech should be punished: philosophers, legislators, politicians, activists, and citizens are highly divided on the issue. The persisting reluctance to sanction hate speech, at least as much as other hate crimes, raises a question for moral psychologists:

Is this lenience ingrained in our moral dispositions, and if so, how?

Linguists and comparative psychologists argue about fine distinctions between verbal and nonverbal actions. Still, most accept that verbal expression is unique amongst other possibly communicative behaviours, such as gestures, facial expressions, or bodily actions: It is the most effective and nuanced way to express mental states, including feelings, abstract ideas, or hypotheticals, and it may be uniquely able to communicate complex information. From a legal perspective but also based on speech uniqueness, the American Constitution's First Amendment (1791), the Declaration of the Rights of Man and the Citizen (1789) and the Universal Declaration of Human Rights (1948) enshrined the Freedom of Speech principle to protect the intrinsic value of speech as a means for free expression and discussion [*Due to the commonness of the phrase “Freedom of Speech”, we will refer to “speech” in what follows, but mean this in a way that includes all language, whether spoken, signed or printed. We also intend “nonverbal” to exclude all uses of such linguistic forms.*].

However, beyond those historical and traditional considerations, this distinction between verbal and nonverbal actions is not a foregone conclusion. Speech can have consequences and cause harm no less than nonverbal actions do^5–12^—granting that harm definition does not reduce to the classic notion of pain as tissue damage^13,14^. The comparison between the negative impact caused by verbal and nonverbal actions does not depend here on how much neural overlap there is between the painful experience caused by social rejection or exclusion and physical damage to the body^15^. Negative consequences for the individual are usually captured by a folk concept of harm, deployed in moral judgment, which is certainly broader than the concept of “pain” in a sense that is tied to a specific neural activation^16^. In addition, the legal concept of harm, distinguished from the mere offence, is broader than actual or possible tissue damage. As argued amongst legal scholars, harm can also include negative consequences for one’s well-being caused by speech^17^, expanding the notion of harm used by Mill nearly two centuries ago^18^.

Depending on the context, harm caused by speech could be as permanent, long-lasting and intense as that caused by nonverbal actions^19,20^. Studies conducted with victims of hate speech and verbal abuse have found high levels of distress and severe psychological damage due to exposure to humiliating, demeaning or discriminatory speech^21–24^. Having made this clear, we should ask whether third-party observers would evaluate verbal and nonverbal hate attacks similarly when both share the same intention to harm others and similar consequences, and if so, why.

The need for answers to these questions is clear when the evidence about moral attitudes towards harmful speech remains equivocal and non-specific. Relevant nationwide surveys on either side of the Atlantic continuously report many citizens who oppose hate speech bans despite believing that harmful discourses are morally unacceptable^25–27^. In the same line, college-student survey respondents broadly support free speech but increasingly favour restrictions on discourse that targets minority groups^28,29^. However, looking at relevant official reports on hate speech, the high proportion of under-reported incidents is striking^30–35^. Does this discrepancy between the increasing readiness to recognise speech as a crime and the lack of reporting hate speech incidents to authorities partly derive from a feature of our moral psychology? Are we less inclined to punish and denounce those who use words rather than physical actions to harm others, and if so, why?

Looking at the related literature in moral psychology, while moral evaluation and punishment rest on cognitive and emotional processes^36–39^, dominant dual-system theories frame such evaluation as weighing the intention and the consequences of the action^40–43^. The exact equivalence in moral condemnation judgement is predicted by theories that argue that our punishment heuristics are driven mainly by outcomes^44^. Granting that verbal (hate speech) and nonverbal hate attacks share similar demeaning and harmful intentions, the explanation from the dominant dual-system theories needs to be that people evaluate the consequences of verbal and nonverbal attacks differently.

Against this consequentialist prediction, we reckoned that the comparative leniency toward hate speech comes not from minimising its harmful consequences but from the tendency to see verbal actions involving speech as inherently less morally negative than nonverbal actions involving the body. Our prediction here extends and complements a range of findings showing that moral evaluation and punishment is determined not only by intentions and consequences but also by associations with some properties of the actions themselves and people’s aversion to them^45–49^.

Miller, Cushman and Hannikainen define this “action aversion” as one’s aversion to intrinsic action’s properties. Together and separately, they found robust support for its importance in first-person and third party moral evaluations^49^. They also showed that action aversion can predict harm condemnation in the context of moral dilemmas, where an affective response to victim suffering (outcome-aversion) can not^47^. For instance, although the moral status of an action (e.g., Lying is wrong) is usually assessed along with its expected consequences (e.g., Lying will cause harm), some typically harmful behaviours (e.g., pushing a person off the footbridge in the so-called trolley problem) might be considered morally worse than atypically harmful ones (e.g., flipping a switch). Even when both lead to the same harmful consequences (e.g., the death of a victim). Experimental evidence also points in the same direction when it demonstrates that people are averse to performing pretended harmful behaviours (e.g., hitting a baby-doll or firing a toy gun towards a friend), even when they are aware of their harmlessness potentiality^45,46^.

Following those findings, we conducted online a vignette-based study. On it, participants assessed verbal and nonverbal hate attacks in which derogatory intent and consequences for the victim (either negative or nonexistent ones) remained equal. We predicted that irrespective of perceiving that both attacks inflict similar harm on their victims, participants would punish and denounce more leniently those committed using words.

Given the lack of a consensus definition of hate incidents and hate speech in the literature, to delimit the scope of our study, we characterise hate actions as ones performed by a perpetrator with a degrading and discriminatory intention towards a victim, based on a particular personal characteristic of the latter (race or ethnic origin, religion, gender, physical or mental conditions, among others). We deliberately avoid stories representing either extreme verbal violence (slurs and death threats) or nonverbal one (punching, beating or kicking), given that vignettes about such actions may distress participants overly, and they are rarely controversial regarding the obligation to denounce them. Moreover, hate speech incidents are less about insults and more about demeaning and discriminatory discourse targeting members of minority or disfavoured groups or identities. Such incidents convey a symbolic message to victims that they are unwelcome and unworthy of social respect^21,50^. Therefore, in our study, participants assessed generic linguistic expressions that also convey harm (e.g. “Go back home!”, or “We don’t want your kind here!”) and nonverbal degrading and discriminatory behaviours (e.g., spitting close to someone’s feet, or stopping someone from sitting next to one).

We conducted the study with native English speakers from the UK. The British legal system has pioneered the implementation of strict hate speech regulations in Western Europe, dating back to the seventeenth century, and the UK is currently the European country that invests the most economical and human resources in combating hate speech and creating social awareness about verbal harm^34,51^. Therefore, if a more lenient approach to hate speech is confirmed even for people whose national legislation reinforces the idea that speech can be as harmful as nonverbal actions, this would bring more confidence for future replications.

Finally, the same concern of scope delimitation underlines the option in favour of testing only a single bias behind all hate-attack scenarios, religious hatred, which is remarkably consistent across countries^31,32^. Since hate attacks are highly context-dependent, we seek this way to minimise the influence that a greater aversion to a specific bias may have on our results. More importantly, in the UK, racially or religiously aggravated offences are, by definition, hate crimes, and just over half (53%) of hate crime’ offences are recorded as one of these racially or religiously aggravated ones^52^. Therefore, race or ethnicity and religion have particular relevance to our study. In addition, they frequently overlap^35,53^. However, while racial or ethnic bias could be linked with several victim profiles, just under half (45%) of religious hate crime offences were targeted against Muslims^52^. Thus, we have chosen to focus on religious hatred against Muslims rather than racial hatred for the present study, as the latter would potentially introduce greater unexplained variance. However, in further studies, we will seek to answer whether our results are replicable with different hate biases (e.g. hatred based on race) or groups of participants (e.g. Americans instead of British).

Our study provides two timely contributions to the literature:

First, the extensive literature in moral psychology on blame and punishment mainly focuses on cases of physical pain^40–43^ ; where damage is caused to someone’s bodily integrity (e.g., killing, wounding) or monetary gains^54^. As words do not overtly or directly cause physical harm or affect economic gains, they are not considered by most literature on third-party punishment and moral judgments, with very few exceptions (e.g., Swim et al.^55^). And, to implement hate speech laws and assure their effectiveness, it is crucial to test whether we could or could not replicate the findings on blame and punishment of physical harm, on speech harm. Moreover, to our knowledge, our study is the first to experimentally apply the action-aversion principle to explain Hate Speech, filling this gap in the literature.

Second, several researchers have called for a better contextualisation of moral psychology^56^. Our study contributes to this agenda by testing scenarios which avoid describing extremely violent aggressions, which are exceptional and focusing on more common demeaning and derogatory actions that victims often encounter. At the same time, it accounts for the role of identities in hate bias—moving away from the psychology of “raceless, genderless strangers”^57^. While we are only testing one type of bias in this study, as we focus on the psychological mechanisms underlying moral leniency towards hate speech attacks, follow-up studies should confirm and extend our findings to different hate biases, social identities, and groups of participants.

Against this background, we formulated **two research questions:**

The first one (**H1**): **Do lay observers evaluate attacks that share the same hate intent and create similar negative consequences for the victim differently, depending on whether they are perpetrated through verbal or nonverbal actions?** And going one step forward, the second **(H2)**: Do lay observers evaluate attacks that share the same hate intent differently, depending on whether they are perpetrated through verbal or nonverbal actions, **even when they create no consequences for the victim**?

And, based on our pilot study, we hypothesized that -consequence and hate intent being the same- participants would be less inclined to punish and denounce hate attacks committed by verbal actions than nonverbal ones, irrespective of consequence type (negative or nonexistent). Additionally, we predicted that participants would rate that both attacks inflicted similar harm on their victims. Our pilot study (N = 171) provided confirmatory hypothesis results (See Stage 1 Registered Protocol): When both actions had the same hate-intent, participants assigned less punishment to verbal actions than to nonverbal ones, yet they rated both types of attacks as comparably harmful. This last finding was confirmed by Bayesian analysis (See Supplementary Information for pilot study details). Our pre-registered hypotheses are documented below:

### H1

Participants will evaluate attacks that share the same hate intent and create similar negative consequences for the victim differently, depending on whether they are perpetrated through verbal or nonverbal actions: More leniently in terms of punishment and denunciation while considering both types similarly harmful.

### H1.a

Participants will **punish verbal hate actions less** than nonverbal ones.

### H1.b

Participants will be **less likely to denounce verbal hate actions** than nonverbal ones.

### H1.c

Participants will rate **verbal and nonverbal hate actions as similarly harmful** to the victim.

### H2

Participants will evaluate attacks that share the same hate intent and create no consequences for the victim differently, depending on whether they are perpetrated through verbal or nonverbal actions: More leniently in terms of punishment and denunciation while considering both types similarly harmful.

### H2.a

Participants will **punish verbal hate actions less** than nonverbal ones.

### H2.b

Participants will be **less likely to denounce verbal hate actions** than nonverbal ones.

### H2.c

Participants will rate **verbal and nonverbal hate actions as similarly harmful** to the victim.

We defended that third-party lower moral condemnation of hate speech would be better explained by action aversion (response to intrinsic action’s properties and their typically associated consequences, irrespective of their actual outcomes) than outcome aversion (response to action’s actual consequences for the victim). Please note that we did not deny the role of outcome aversion in moral condemnation by no means. Instead, we defended the idea that when ordinary citizens face verbal and nonverbal hate attacks, the action aversion against verbal ones would be significantly lower independently of their consequences. We suggested that something intrinsic in words and speech, traditionally and historically linked with legitimate informational and cooperative purposes, would make people more likely to grant them special protected status and be more lenient towards their harm.

## Methods

(See Supplementary Information for Testing Materials).

### Study description

The study is an online experiment based on the contrastive vignette method. A 2 × 2 mixed design was implemented with the following independent variables (IV), the first, **action type**, as a within-subjects factor with two levels: verbal and nonverbal hate-attacks, and the second, **consequence type**, as a between-subjects factor with two levels: negative consequence and nonexistent consequence for the victim. In addition, participants’ ratings of three dependent variables (DV) were collected in random order: Appropriate punishment, the likelihood of denouncing perpetrators to competent authorities, and the level of harm inflicted on the victim.

Participants, as lay-observers, contrasted situations where a character with the same degrading intention against a targeted victim performs either a verbal attack (using words) or a nonverbal (bodily) one. The description of the consequence for the victim in both cases was explicitly the same (See Supplementary Information section for Testing Materials).

Additionally, to better test the action aversion theory (which predicts that moral judgments are driven by one’s aversion to intrinsic action’s properties and their typically associated consequences, irrespective of their actual outcomes), we allocated participants randomly into two groups. Group A participants tested two experimental trials, verbal and nonverbal hate actions with identical negative consequences for the victim (e.g., As a consequence of the hate attack, the victim who suffered it stops using the bus line in which he was attacked). Group B participants also assessed two experimental trials, but this time, with no consequences for the targeted victims (e.g., we let participants know that the victim was deaf and could not hear the hate speech remark).

Participants in Group A were presented with six vignettes in random order: The two experimental trials, verbal and nonverbal, three distractors, and an attention check: 1)Verbal hate action based on religious hatred with negative consequences for the victim, 2) Nonverbal hate action based on religious hatred with negative consequences for the victim, 3) Distractor 1: Neutral action in a religious hatred context, 4) Distractor 2: Verbal hate action against meat eaters, 5) Distractor 3: Nonverbal hate action against environment polluters, 6) Attention check. Participants in Group B were also presented with six vignettes in random order. Still, in this case, both verbal and nonverbal scenarios had no actual consequences for the victim: 1)Verbal hate action based on religious hatred with nonexistent consequences for the victim, 2) Nonverbal hate action based on religious hatred with nonexistent consequences for the victim, 3) Distractor A: Neutral action in a religious hatred context, 4) Distractor B: Verbal hate action against meat eaters, 5) Distractor C: Nonverbal hate action against environment polluters and 6) Attention check.

**Testing Materials** (See Supplementary Information for the Study Testing Materials).

#### Experimental vignettes

All experimental vignettes shared a similar structure: Scenario setting (1, 2 or 3), description of the perpetrator's hostility towards a specific group (Muslims) to which the victim belongs (4), an opportunity to convert that hostility into action (5), the performance of a hate-attack (6 or 7), and a final outcome (8 or 9).

Participants were randomly assigned to one of the three scenario settings (1: Bus, 2: Train or 3: Supermarket) and, within that scenario, allocated into two groups, where the IV consequence type was manipulated: Participants in group A received two experimental trials (verbal and nonverbal) with negative consequences for the victim as the same final outcome (8). Participants in group B received the two experimental trials (verbal and nonverbal) with nonexistent consequences for the victim as the same final outcome (9). Aspects (4) and (5) remain broadly similar across vignettes. Finally, we manipulated the IV action type in the action's performance, being either verbal (6) or nonverbal (7).

An example of a nonverbal action was 'John looked Bilal in the eyes and spat on the ground next to him', while an example of a verbal action was 'John yelled at him, "Go home! Stop Islamization of our country!"'. (See Supplementary Information for the Study Testing Materials).

#### Distractors

Three distractors were presented to participants. The inclusion of distractors in this study served two objectives: 1) To make the study goals less evident, 2) To maintain participants' attention. The testing vignettes were similar in structure and background settings, and without distractors, it could have been easy for participants to detect the study's aim. In addition, the repetitive scenes might have reduced participants' interest and attention, which lead to haphazard responses^58^.

The structure of the distractor vignettes was the same as the testing vignettes'. However, their differences lie in the hostility towards the victim or the type of action performed by the perpetrator. In some distractors, instead of a hate action which causes the victim either negative consequences or nonexistent ones, a non-relevant or neutral action was involved (e.g. 'John briefly glanced at his watch to check the time and continued reading his book.'). In some others, the hostility shown was not related to religious hatred (the current interest of the study). Instead, the perpetrator's hostility was based on the victim's food or lifestyle preferences (i.e., hostility from a pro-animal activist against a meat-eater). An example of a distractor vignette read as follows (See Supplementary Information for Study Testing Materials):*' In the supermarket, Emma saw Anna putting some salami and pork rib in her shopping trolley. Emma is vegan and strongly opposes the act of killing and eating animals. As Emma walked past Anna, she shouted at her, 'I wish you suffocate to death with your salami!'. As a consequence, Anna stopped going to the supermarket alone.'*’

#### Attention task

An attention task appeared randomly throughout the experiment to ensure participants read both vignettes and instructions. The attention task involved a vignette-format text but included instructions to direct participants' ratings in the relevant questions. Participants had to rate the questions as instructed to pass the attention check. An example of an attention task read as follows: *'This is a test for us to make sure that you read all the scenarios very carefully. Please answer the following questions, rating to what extent you think Mary should be punished as "Not at all (0)"; while rating how harmful Mary's action was as "Very Much (6)"’*.

### Procedure

We conducted the study using the Qualtrics software (www.qualtrics.com). After eliciting informed consent at the start, all vignettes, distractors, and attention tasks were presented randomly to the participants.

The study measured three dependent variables: Appropriate punishment, the likelihood of denouncing perpetrators to competent authorities, and the level of harm inflicted on the victim. Participants were presented with relevant questions and asked to rate their responses on a 7-point, forced-choice, continuous Likert scale, ranging from 0 (not at all, nothing at all or very unlikely) to 6 (very much, very likely, or extremely). With the forced-choice scale, participants had to process each question and provide a response^59^ to proceed to the next one or the following vignette.

At the end of the experiment, demographic details (age, sex, educational background, degree of religiousness, social and political ideology and whether they have ever suffered a discriminatory experience) were collected. Finally, the last question is presented to gauge participants' awareness about legal sanctions against hate attacks in their country of residence (i.e. Are hate incidents legally sanctioned in your country of residence?). Data collection was blinded since experimenters had no contact with the participants.

### Pre-registered sampling plan, power analysis and exclusion criteria

Participants were recruited through Prolific (https://prolific.co), an online testing platform considered a reliable source of data collection^60^, which provides the flexibility to expand the range of people and geographical areas that can be included in the sample^61^. This was especially useful since our study recruited only participants who currently live in the U.K. and have English as their first language.

For hypotheses H1a, H1b, H2a, and H2b, an a priori power analysis conducted using G-Power software^62,63^ showed that a total of 1302 participants was needed to run a mixed within-between-subjects MANOVA with sufficient power (alpha at 0.05, power (1 − β) set to 0.95, effect size f (V) set to 0.1, two groups and two measures). Furthermore, since H1c and H2c assumed no significant differences in participants' scores, no minimal sample size was required to test those hypotheses.

We set recruitment parameters in Prolific to only choose individuals whose first language was English, ensuring that all participants fully understood the vignettes presented and avoiding possible misunderstanding of the vignettes and/or instructions given. In addition, incomplete and duplicate submissions were manually excluded too. Only submissions with a valid Prolific ID, which anonymously refers to a unique participant, were approved. Based on Prolific ID, we excluded duplicate submissions except for the initial one if it was complete and did not coincide with another submission by the same participant. Finally, participants who failed the attention check or took more than 15 min to finish with all the questions presented were excluded. Consequently, from a total of 1403 participants that were recruited, 1309 remained after applying the exclusion criteria mentioned above, complying with the sample size of the a priori power analysis.

### Pre-registered analysis and results

Data were pre-processed by applying the exclusion criteria mentioned above. Since our pilot study showed a correlation between dependent variables, as first step, we aimed to evaluate differences in participants' mean scores on the three dependent variables (Appropriate punishment for the perpetrator, the likelihood of denouncing perpetrators to competent authorities and the level of harm inflicted on the victim), across levels of the independent factors, namely, action type (as a within-subjects factor: verbal and nonverbal hate actions) and consequence type (as a between-subjects factor: negative consequences and nonexistent consequences for the victim). All data analyses were performed using R (version 4.1.1).

The following assumptions were assessed to run a mixed MANOVA: No multivariate outliers, homogeneity of variance and covariance, multivariate normality, Linearity and Multicollinearity. Multivariate outliers were tested by computing Mahalanobis distance for each observation, and eighteen (18) participants were identified as multivariate outliers (*p* < 0.001) and consequently removed according to the pre-registered protocol. Therefore the final sample size for the analysis was 1291 participants. The homogeneity of variance–covariance matrices was assessed via Box's M test and the assumption of variance was violated (results showed *p* < 0.05). Therefore, as pre-registered, this violation was further investigated using Levene's test for multiple independent variables. Again it showed violations (*p* < 0.05). Multivariate normality was checked using Mardia's skewness and kurtosis test, and violations were found (*p* < 0.05). Linearity was assessed with scatterplots, and it was present. Finally, multicollinearity was tested for the three dependent variables and was not observed. No correlation was above r = 0.90^64^. Since the assumptions required for the MANOVA were violated, a Johansen's^65^ general formulation of Welch-James's statistic with Approximate Degrees of Freedom (ADF), which is suitable for non-parametric mixed designs, was applied^66–68^ to evaluate differences in participants' mean scores on the three DV across the IVs. The results are reported in WJ format (df1, df2) for the Welch-James ADF statistic tests. The df1 and df2 are the approximate degrees of freedom for the numerator and denominator. Only results of *p* < 0.05 were considered statistically significant.

The Welch-James ADF test showed a significant main effect of the IV action type (WJ (3, 1041) = 351.69, df = 1041, *p* < 0.001), a significant main effect of the IV consequence type (WJ (3, 1026) = 295.00, df = 1026, *p* < 0.001), and a significant interaction between action type and consequence type on the combined dependent variables (WJ (3, 1041) = 14.32, df = 104, *p* < 0.001). Therefore, according to the pre-registered protocol, to further investigate those findings, a post hoc analysis was conducted for each dependent variable respectively.

Again, the following assumptions were previously tested: No outliers, normality, homogeneity of variance, and homogeneity of covariance. First, the Box Plot method was used to detect outliers, but none were found. Then, the data were analysed via histograms and Q-Q plots to test for normality, and the assumption was violated. Next, the homogeneity of variance was assessed via dot plots and Levine’s test and it was violated (all results were *p* < 0.05). The homogeneity of covariance was evaluated via Box’s M test, and it showed significant results for harm (*p* < 0.001) but not for punishing (*p* = 0.105) nor for reporting (*p* = 0.992). In addition, the assumption of sphericity was taken for granted since there were only 2 within-subjects levels. Since the assumptions required were violated, univariate testing for each of the three DV was conducted using the Holm-corrected Welch ADF test instead of using three mixed ANOVAs.

**Figure 1:**
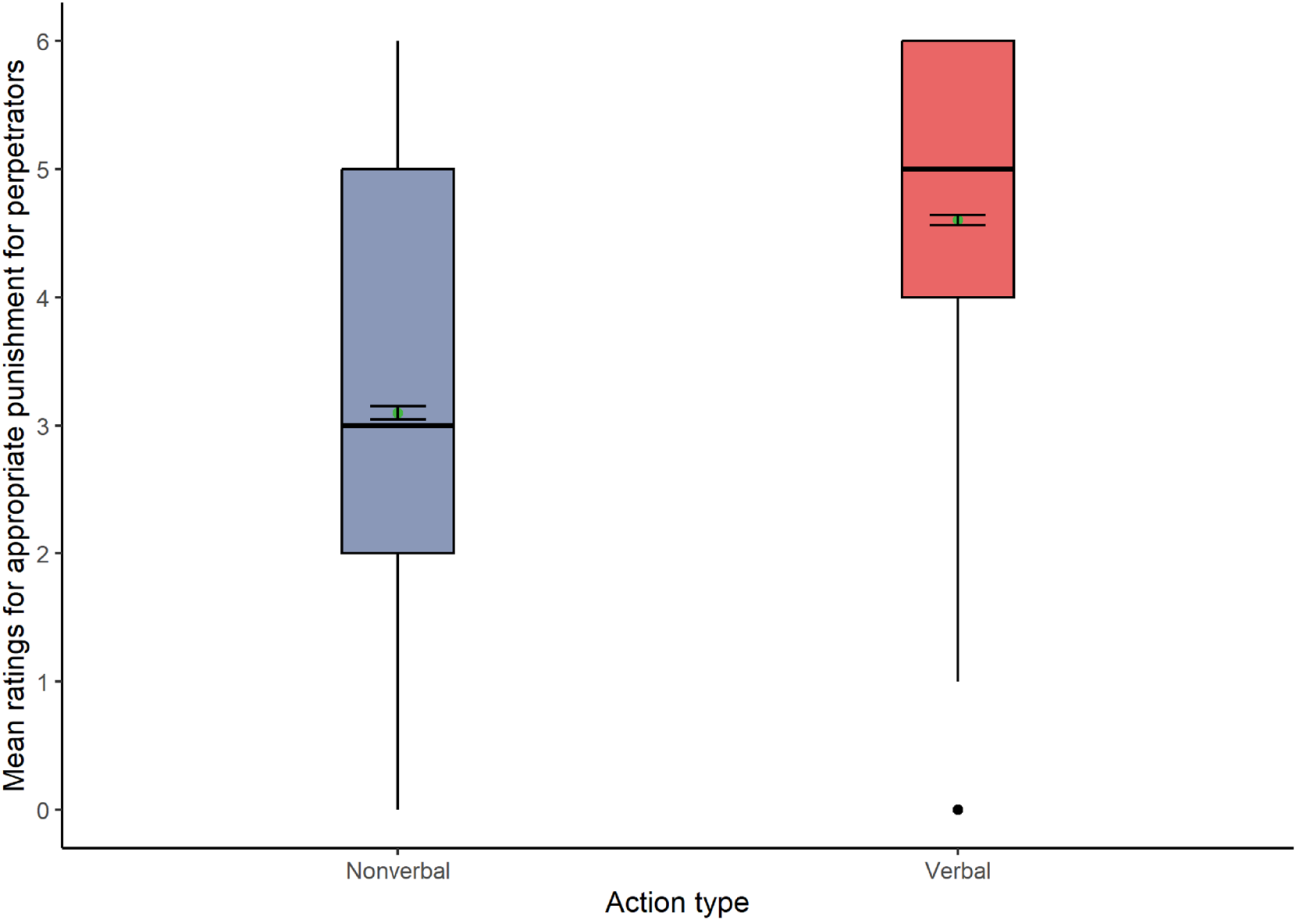
Box plots show the appropriate punishment for each action type (N = 1291).

As predicted, the first Holm-corrected Welch-James ADF test for the dependent variable appropriate punishment showed a significant main effect of action type (Fig. 1). However, **H1a** and **H2a** were not supported since the analysis showed higher mean scores for verbal (Mean = 4.60) than nonverbal hate actions (Mean = 3.10): WJ (1, 1287) = 938.99, *p* < 0.001.

**Figure 2:**
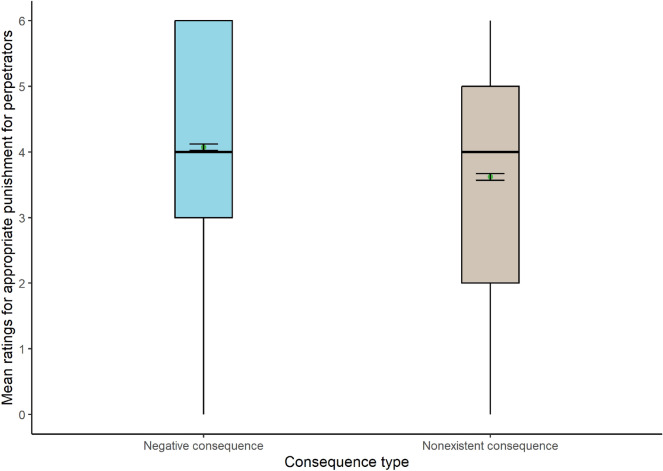
Box plots show the appropriate punishment for each consequence type (N = 1291).

The analysis also showed a significant main effect of consequence type (Fig. 2), with higher mean scores for negative (Mean = 4.07) than nonexistent consequences for the victim (Mean = 3.62): WJ (1, 1275) = 32.152, *p* < 0.001. No interactions between the two IVs were found.

**Figure 3:**
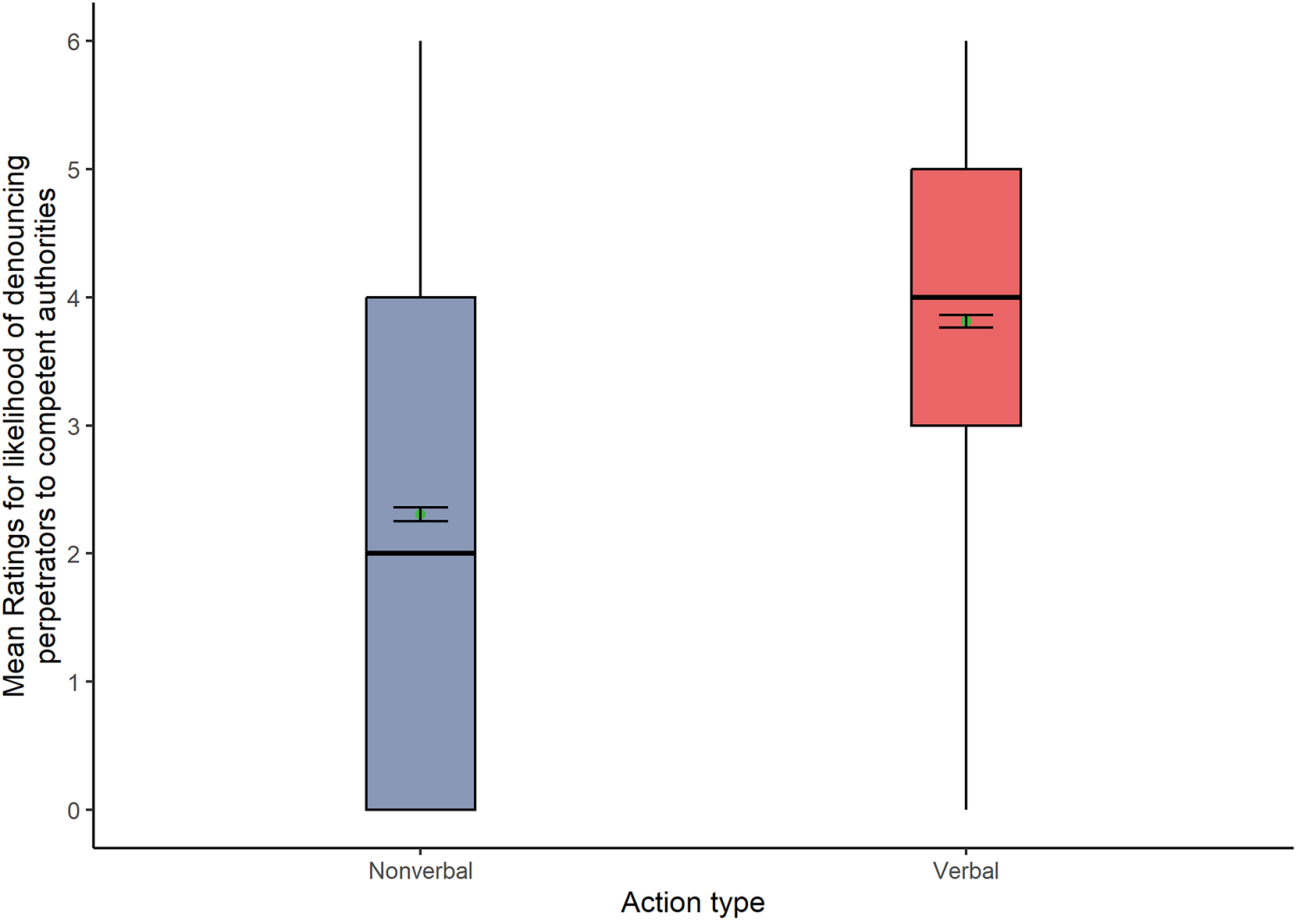
Box plots show the likelihood of denouncing perpetrators for each action type (N = 1291).

The second Holm-corrected Welch-James ADF test for the dependent variable likelihood of denouncing perpetrators to competent authorities showed a significant effect of action type (Fig. 3), with higher mean scores for verbal (Mean = 3.81) than nonverbal actions (Mean = 2.31): WJ (1, 1286) = 797.64, *p* < 0.001. Therefore, **H1b** and **H2b** were unsupported.

**Figure 4:**
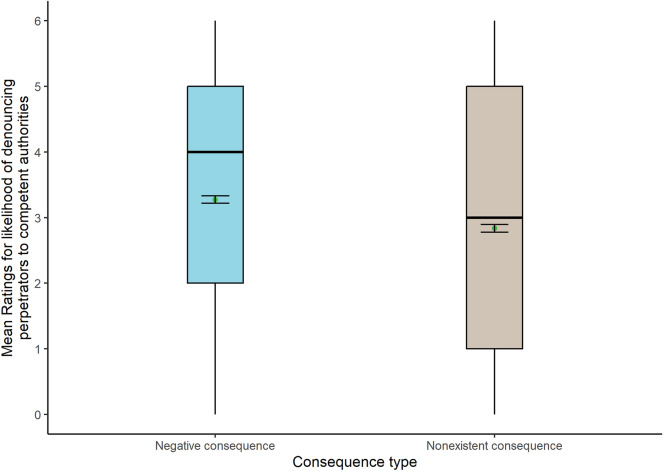
Box plots show the likelihood of denouncing perpetrators for each consequence type (N = 1291).

The analysis showed a significant main effect of consequence type also for this dependent variable (Fig. 4), with higher mean scores for negative (Mean = 3.27) than nonexistent consequences for the victim (Mean = 2.84): WJ (1, 1286) = 22.96, *p* < 0.001. Again, no interactions between the two IVs were found.

**Figure 5:**
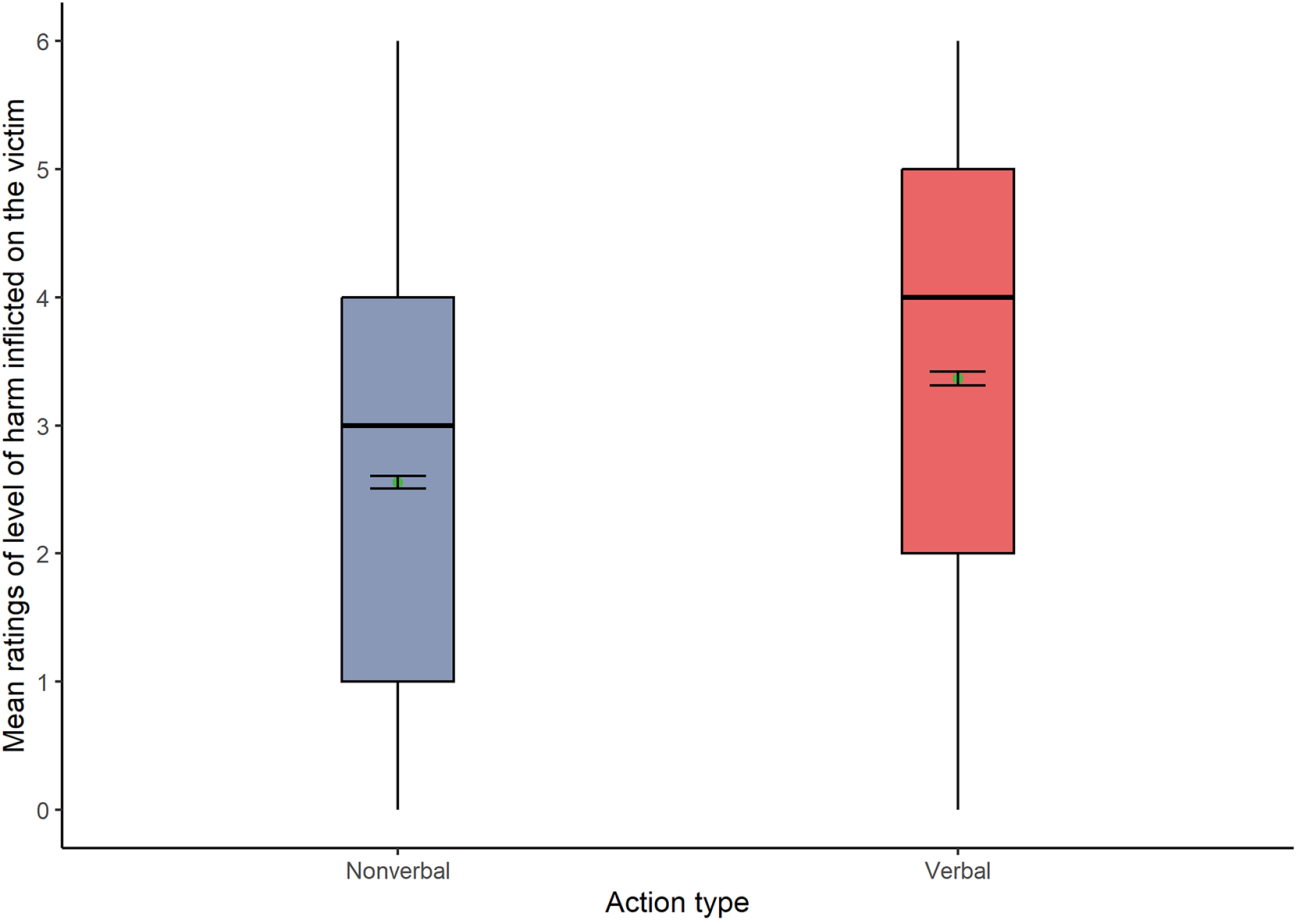
Box plots show the level of harm for each action type (N = 1291).

The third Welch-James ADF test for the dependent variable level of harm inflicted on the victim showed a significant effect of action type (Fig. 5). Therefore, **H1c** and **H2c** were unsupported. Results consistently showed higher mean scores for verbal (Mean = 3.37) than nonverbal actions again (Mean = 2.55): WJ (1,1240) = 348.78, *p* < 0.001.

**Figure 6:**
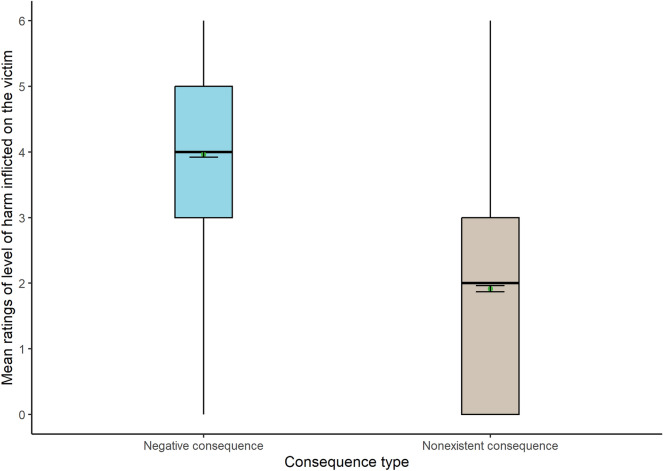
Box plots show the level of harm for each consequence type (N = 1291).

In addition, results confirmed a significant main effect of consequence type (Fig. 6), with higher mean scores for negative (Mean = 3.96) than nonexistent consequences for the victim (Mean = 1.91): WJ (1, 1227) = 763.80, *p* < 0.001.

**Figure 7:**
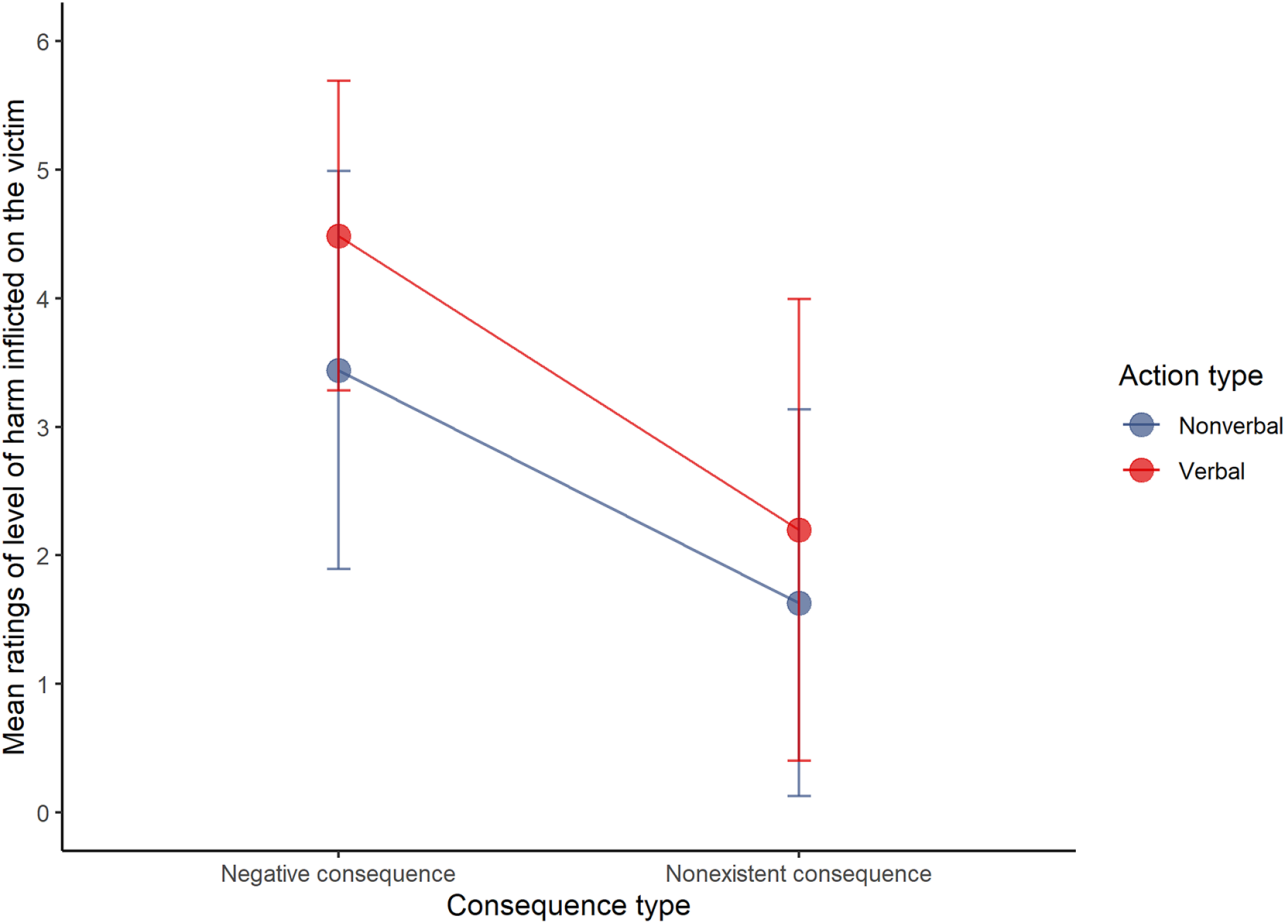
Interaction plot between action type and consequence type in the level of harm inflicted on the victim (N = 1291).

Finally, a significant interaction (Fig. 7) between consequence type and action type was found: WJ(1, 1240) = 30.83, *p* < 0.001. As pre-registered, since the data were ordinal, a Games Howell post hoc testing was conducted (instead of the Tukey method) to further analyse the interaction (Tables 1 and 2). Results showed significant differences between all groups (*p* < 0.001).

**Table 1:** Results of Games–Howell post-hoc test grouped by action type.

Action type	Group1	Group2	n1	n2	Estimate	CI	SE	Statistic	df	*p* value
Nonverbal	Nonexistent consequence	Negative consequence	632	659	1.79	[1.62–1.93]	0.06	21.05	1288.70	< .001
Verbal	Nonexistent consequence	Negative consequence	632	659	2.27	[2.10–2.44]	0.06	26.50	1098.37	< .001

**Table 2:** Results of Games–Howell post-hoc test grouped by consequence type.

Consequence type	Group1	Group2	n1	n2	Estimate	CI	SE	Statistic	df	*p* value
Nonexistent consequence	Nonverbal	Verbal	632	632	0.57	[0.38–0.75]	0.06	6.11	1224.09	< .001
Negative consequence	Nonverbal	Verbal	659	659	1.05	[0.9–1.20]	0.05	13.76	1243.59	< .001

### Exploratory analysis and results

Consistent with our Stage 1 Registered Protocol, we ran an exploratory analysis to examine whether individual differences in sensitivity to verbal actions would predict differences in moral judgements of verbal hate attacks (See Supplementary Information for testing materials, analysis and results). We found positive, statistically significant, and medium correlations between ratings of sensitivity to verbal actions and ratings in deserved punishment, likelihood to denounce and the level of harm of verbal hate actions. Moreover, regression analyses showed that ratings of sensitivity to verbal actions significantly and positively predicted ratings in deserved punishment, likelihood to denounce and harmfulness of verbal hate actions. However, the questionnaire and the rating scale were not previously validated to test sensitivity to verbal actions. Therefore we consider our results inconclusive and that a more rigorous scale and testing materials are needed to further investigate possible links.

In addition, to analyse the data collected further, we explored possible interactions with participants' demographics (age, sex, educational background, degree of religiousness, social and political ideology, previous discriminatory experiences and the awareness of legal consequences for hate incidents), which are reported as supplementary material.

### Ethics information

The study complied with all ethical regulations. Ethics approval was obtained from the local Ethics Committee at the LMU (ID-Number 131874). Participants provided informed consent at the outset of the experiment, and all received relevant information about the research aim, procedure, duration, and compensation. They also were informed that no expected risk would be involved by taking part in the experiment and about the option of withdrawing from the study at any time without ensuing consequences. Participants were compensated with 1.20 pounds for 10 min of participation.

## Discussion

The high proportion of underreported hate speech attacks, the relatively few cases that end with sanctions, and the inconclusive evidence about moral attitudes towards these harmful incidents, made us wonder whether that lenience towards speech harm was ingrained in our moral dispositions, and consequently made us evaluate the harm caused by words as less harmful and worthy of punishment and denunciation than equivalent physical damage. Therefore, in an online 2 × 2 factorial experiment (N = 1309) based on the contrastive vignette method, we tested action type and outcome aversion and its interaction in participants' evaluations of verbal and nonverbal hate incidents driven by the same hate intent and which create the same consequences for the victims.

Following the principle of action aversion, which explains that moral evaluation and punishment are determined not only by intentions and outcomes but also by associations with some intrinsic properties of the actions and people's aversion to them, we predicted that people would evaluate verbal and nonverbal hate-intended actions differently, being more lenient with verbal ones, even when both create the same consequences for the victims.

Expectedly, participants assessed hate actions with a negative outcome for the victim more severely than those that did not succeed in that purpose. In addition, they did evaluate verbal and nonverbal hate actions differently, irrespective their outcome. Therefore, our results provide evidence supporting both main effects, action and outcome aversion, in people's ratings of deserved punishment, denunciation and the level of harm inflicted on the victim, and a significant interaction of both effects in the last. However, contrary to our predictions, participants in our study consistently rated verbal hate actions as more worthy of punishment and denunciation and more harmful than nonverbal ones.

One possible explanation for these results could be that participants have found the hate intent more evident in hate speech than in other hate-motivated attacks. For example, the disdain and contempt towards the victims could be more explicit in verbal hate attacks, exacerbating participants' moral condemnation. Additionally, it could be the case that the harm in nonverbal attacks that do not reach the extreme of kicks and punches can be more plausibly denied by offenders and observers, providing them with moral wiggle room to remain inactive. Moreover, compared to a nonverbal hate attack, a verbal one has a host of related harms that go beyond the damage inflicted on the victim: that caused to bystanders, to other members of the targeted collective, and to society as a whole, which folk intuitions could capture.

Another possible explanation is that participants could have overestimated the degree to which a discriminatory comment would provoke their rejection. This would explain why the proportion of under-reported hate speech attacks continues to be striking despite participants self-report more severe evaluations of verbal hate actions in terms of punishment, denunciation and harmfulness. As pointed out by Kawakami et al.^69^, ordinary citizens usually fail to predict how they would feel and behave when faced with an act of racism. In their study, participants indicated that they would be very upset when witnessing such an incident, but they finally showed little emotional distress and responded indifferently. Therefore, although participants in our study reported they would be more severely against verbal hate actions, they may have wrongly anticipated their responses in real life. Further research is needed to test these possible explanations for a more severe response to harm caused by words.

### Differences with pilot study results

Two additional reasons could help to explain why we did not replicate the findings from our pilot study, in which participants assessed nonverbal hate attacks more severely than verbal ones while considering both as similarly harmful.

First, the pilot study tested a different version of scenarios A1 and A2. In them, the perpetrator, the victim, the perpetrator's hate intent, and the verbal and nonverbal actions were the same, but we avoided mentioning the consequences to participants. Recently, Kneer and Skoczén^70^ showed that the folk concept of punishment is outcome-dependent. Furthermore, as they pointed out, prominent studies^71,72^ showed that learning about negative consequences can influence people's assessments of ethicality, to the point of assessing behaviours previously considered acceptable, as more unethical after being told about their consequences. In addition, as has been empirically demonstrated^73^, in some cases, the simple consideration of alternative outcomes could alter participants' judgements. As a result, it is possible that introducing information about the consequences created for the victim affected participants' judgements, principally of deserved punishment and the likelihood of denouncing perpetrators. Therefore, in future work, we plan to specifically explore the effect of mentioning and not mentioning the action's negative consequences on participants before asking them to assess harmful verbal and nonverbal hate actions.

Second, another critical factor that could help to explain the variation between pilot and pre-registered study results is that both were tested during the pandemic of COVID19, but in totally different circumstances. On the one hand, the pilot study was conducted on November 2020, at the height of a global pandemic, with burdensome restrictions to prevent the spreading. And it tested a single root scenario, whose nonverbal version described a perpetrator spitting close to the victim's feet. Possibly, we underestimated the role of disgust in moral judgement in those circumstances. As some experts defend^74^, disgust is thought to have evolved as a biological mechanism that puts distance between us and anything that could potentially infect us. Therefore presenting a scenario where someone spits close to the victim could have distressed participants overly, even more if they considered that the perpetrator might have removed his mask in doing so, an action that was expressly forbidden at that time. On the other hand, the pre-registered study was tested on August 2022, when most people were fully vaccinated, and the rules of using masks and keeping their distance were lifted. This time the study also tested several verbal and nonverbal scenarios, one of the nonverbal described a perpetrator stopping the victim from sitting next to him in public transport, which at that time was normalised as a measure that helps to control the spreading. As a result, it could be possible that the nonverbal action of spitting close to the victim's feet was assessed more severely in the pilot study than in the pre-registered one. Again, additional investigations are needed to test these explanations for a more severe response to harm caused by words.

Finally and in addition to the above, our results replicate, for verbal (speech) harm, the moral luck phenomenon tested by Kneer and Skoczén^70^ in a recent study with implications for social psychology and moral theories. This phenomenon makes people assessing potential harm as more likely when it does come to pass than when it does not and, therefore, they judge unlucky perpetrators (whose actions ended in adverse outcomes) more severely than lucky ones (whose actions were neutral or ended harmlessly due to an external event). In our study, participants were randomly distributed into 2 groups: Those in group A tested two experimental trials (verbal and nonverbal), ending with the same negative consequences. Participants in group B tested the same experimental trials. Still, this time the victim luckily did not suffer the expected consequences due to an external event (e.g., the victim was deaf and could not hear the hateful remark or was distracted and did not see the perpetrator's reaction). Our results showed that participants judged the unlucky perpetrators more severely than the lucky ones in terms of punishment, denunciation and the level of harm inflicted on the victim.

## Conclusion

Our results show that people are more averse towards hate-motivated verbal actions than physical ones when intention and consequences remain equal. Against our predictions, we demonstrate that "words could hurt more than actions" also holds for third-party observers, who self-reported they would punish hate discourses more than equivalent physical acts. These results extend and complement a range of findings showing third-party' moral evaluation and punishment heuristics are determined not only by intentions and consequences but also by associations with intrinsic actions' properties and people's aversion to them. We confirmed that no lower moral condemnation of verbal hate attacks is ingrained in ordinary people's moral dispositions, with implications for social psychology and moral theories. Moreover, our results show that ordinary citizens are open to recognising the harm caused by words which is a solid basis for developing public policies that reinforce civic engagement against hate speech incidents. Additionally, these findings contribute to the discussion regarding the limits of the freedom of speech principle that set them in the actual harm caused to victims, reporting a folk intuitions assessment of hate speech harms. In sum, our findings confirm that a better understanding of the psychological processes behind the moral condemnation of verbal harm, concretely hate speech, is needed to develop and implement efficient regulations and policies against such harmful discourses.

In this study, we deliberately avoided scenarios representing extreme verbal and nonverbal violence. In this context, our results show that ordinary citizens self-report a higher tendency to punish and denounce demeaning and discriminatory speech targeting members of minority or disfavoured identities than comparable nonverbal actions, which is relevant for legislative and policy efforts against hate speech. Nonetheless, future work could explore the limits of this comparison, contrasting folk intuitions about more extreme scenarios (e.g., comparing the use of slurs and death threats with episodes of punching, beating or kicking). In addition, in the present study, we recruited only native English speakers from the UK since this country pioneered the implementation of hate speech regulations in Western Europe and currently invests the most economical and human resources in creating social awareness about verbal harm. However, hate speech is a growing concern which affects similarly many contemporary democracies around the world. Therefore, further research is needed to explore whether our results are replicable with different groups of participants (e.g. Americans or non-English speaking populations). Finally, we focused here on anti-Muslim hate speech because just over half of the hate-crime offences in the UK are recorded as racially or religiously aggravated. Again, further research could explore whether our results are replicable with different hate biases (e.g. hatred based on race, sexual orientation or physical and mental disabilities).

## Supplementary Information

Supplementary Information.

## Data Availability

The data sets generated and analysed for this study are available through the Open Science Framework: 10.17605/OSF.IO/WBASX.

## Code availability

Custom code that supports the findings of this study is also available through the Open Science Framework: 10.17605/OSF.IO/WBASX.
