# Supplementary material for: Ordinary citizens are more severe towards verbal than nonverbal hate-motivated incidents with identical consequences
Source: Sci Rep. 2023 May 2;13:7126. doi: 10.1038/s41598-023-33892-8 (PMC10154298; doi:10.1038/s41598-023-33892-8)
Supplement: Supplementary file 1 — Supplementary Information. [file 41598_2023_33892_MOESM1_ESM.pdf]

Supplementary Information: Appendix 1

Study Testing Materials (Main and exploratory studies)

MAIN STUDY (Experimental trials)

Scenario setting A: Bus

Nonverbal A1 with negative consequences

Bilal, a Muslim man, was coming home by bus after prayers at the mosque. Peter was another passenger on the bus who had often shown an intolerance of Muslims. He had never met Bilal but saw him leaving the mosque before getting on the bus. This bus was the only public transport between the mosque and Bilal's apartment, and the walk would otherwise take him an hour. When the bus got to his stop, Bilal had to walk past Peter. Peter looked Bilal straight in the eyes and spat on the floor next to him. As a consequence, Bilal stopped using that bus line for a month.

Verbal A2 with negative consequences

Bilal, a Muslim man, was coming home by bus after prayers at the mosque. Peter was another passenger on the bus who had often shown an intolerance of Muslims. He had never met Bilal but saw him leaving the mosque before getting on the bus. This bus was the only public transport between the mosque and Bilal's apartment, and the walk would otherwise take him an hour. When the bus got to his stop, Bilal had to walk past Peter. Peter yelled at him, "Go home! Stop Islamization of our country!" As a consequence, Bilal stopped using that bus line for a month.

Nonverbal A3 with nonexistent consequences

Bilal, a Muslim man, was coming home by bus after prayers at the mosque. Peter was another passenger on the bus who had often shown an intolerance of Muslims. He had never met Bilal but saw him leaving the mosque before getting on the bus. This bus was the only public transport between the mosque and Bilal's apartment, and the walk would otherwise take him an hour. When the bus got to his stop, Bilal had to walk past Peter. Peter looked Bilal straight in the eyes and spat on the floor next to him. Bilal had very poor eyesight, so he did not notice Peter's reaction.

Verbal A4 with nonexistent consequences

Bilal, a Muslim man, was coming home by bus after prayers at the mosque. Peter was another passenger on the bus who had often shown an intolerance of Muslims. He had never met Bilal but saw him leaving the mosque before getting on the bus. This bus was the only public transport between the mosque and Bilal's apartment, and the walk would otherwise take him an hour. When the bus got

to his stop, Bilal had to walk past Peter. Peter yelled at him, "Go home! Stop Islamization of our country!" Bilal used his wireless headphones and listened to loud music, so he could not hear a word coming from Peter.

Scenario setting B: Train

Nonverbal B1 with negative consequences

Ali, an elderly Muslim man, was returning home by train after a religious festival. Mark was another passenger sitting on the train who had always despised Muslim people. He had never met Ali but saw that he was wearing a Tarbush (A traditional-Muslim red hat). The seat next to Mark was the only one free in the waggon. Ali saw it and was heading to take it, but Mark scowling, put his backpack promptly on the free seat, stopping Ali from sitting next to him. As a consequence, Ali stood the whole journey.

Verbal B2 with negative consequences

Ali, an elderly Muslim man, was returning home by train after a religious festival. Mark was another passenger sitting on the train who had always despised Muslim people. He had never met Ali but saw that he was wearing a Tarbush (A traditional-Muslim red hat). The seat next to Mark was the only one free in the waggon. Ali saw it and was heading to take it, but Mark scowling, yelled at him, "Go where you came from! You are making our country sick!" As a consequence, Ali stood the whole journey.

Nonverbal B3 with nonexistent consequences

Ali, an elderly Muslim man, was returning home by train after a religious festival. Mark was another passenger sitting on the train who had always despised Muslim people. He had never met Ali but saw that he was wearing a Tarbush (A traditional-Muslim red hat). The seat next to Mark was the only one free in the waggon. Ali saw it and was heading to take it, but Mark scowling, put his backpack promptly on the free seat, stopping Ali from sitting next to him. Suddenly, a closer seat was left free, so Ali took it without even noticing Mark's reaction.

Verbal B4 with nonexistent consequences

Ali, an elderly Muslim man, was returning home by train after a religious festival. Mark was another passenger sitting on the train who had always despised Muslim people. He had never met Ali but saw that he was wearing a Tarbush (A traditional-Muslim red hat). The seat next to Mark was the only one free in the waggon. Ali saw it and was heading to take it, but Mark yelled at him, "Go where you

came from! You are making our country sick!" Ali was attentively listening to his favourite audiobook, so he could not hear a word coming from Mark.

Scenario setting C: Supermarket

Nonverbal C1 with negative consequences

Hamza, a Muslim man, was shopping at his local supermarket. Harry was another client at the supermarket who had often shown an immense disdain for Muslim people. He had never met Hamza but saw him asking for halal meat (meat that meets requirements that Muslims consider to make it suitable for consumption). Harry realized that there was only one package of Halal beef left. He looked at Hamza and, smiling derisively, put the package into his shopping cart. As a consequence, Hamza stopped going to that supermarket.

Verbal C2 with negative consequences

Hamza, a Muslim man, was shopping at his local supermarket. Harry was another client at the supermarket who had often shown an immense disdain for Muslim people. He had never met Hamza but saw him asking for halal meat (meat that meets requirements that Muslims consider to make it suitable for consumption). When heading to supermarket check-out, Hamza had to walk past Harry. Harry yelled at him, "Get out! Stop destroying our culture!" As a consequence, Hamza stopped going to that supermarket.

Nonverbal C3 with nonexistent consequences

Hamza, a Muslim man, was shopping at his local supermarket. Harry was another client at the supermarket who had often shown an immense disdain for Muslim people. He had never met Hamza but saw him asking for halal meat (meat that meets requirements that Muslims consider to make it suitable for consumption). Harry realized that there was only one package of Halal beef left. He looked at Hamza and, smiling derisively, put the package into his shopping cart. Hamza was texting on his cell phone and did not notice Harry's reaction.

Verbal C4 with nonexistent consequences

Hamza, a Muslim man, was shopping at his local supermarket. Harry was another client at the supermarket who had often shown an immense disdain for Muslim people. He had never met Hamza but saw him asking for halal meat (meat that meets requirements that Muslims consider to make it suitable for consumption). When heading to supermarket check-out, Hamza had to walk past Harry.

Harry yelled at him, "Get out! Stop destroying our culture!" Hamza was loudly talking on his cell phone, so he could not hear a word coming from Harry.

Distractors:

Distractor 1 (Neutral): Neutral action in a religious hatred context
Ahmed, a Muslim man, was on a flight coming back home after a conference in Morocco. John was another passenger on the flight who had often shown an intolerance of Muslims. He had never met Ahmed but saw him leaving the prayer room at the airport before getting on the plane. When the plane landed and passengers were asked to leave it, Ahmed had to walk past John. John briefly glanced at his watch to check the time and continued reading his book.

Distractor 2 (Nonverbal BMW): Nonverbal hate action against environment polluters
Albert saw James stepping out of a brand-new BMW parked outside an offices' building on his way to work. As an environmental activist, Albert despises luxurious car manufacturers. He considers all car manufacturers should be banned from polluting the environment and worsening climate change. Seeing James leave the luxury car, Albert went forward and kicked the rear fender in front of him. As a consequence, James stopped using that car park for a month.

Distractor 3 (Verbal Vegan): Verbal hate action against carnivores
Emma saw Anna putting some salami and pork-rib in her shopping trolley in the Supermarket. Emma is vegan and strongly opposes the act of killing and eating animals. As Emma walked past Anna, she shouted at her, 'I wish you suffocate to death with your salami! '. As a consequence, Anna stopped going to a supermarket alone.

Attention check:
Daniel is at a restaurant that just opened in his neighbourhood. Because it is a Mexican restaurant, Daniel believes, please ignore the rest of the information for this scenario. This is a test for us to make sure that you are reading all the scenarios carefully. Please answer the following questions, rating to what extent do you think Mary should be punished as Not at all (0); while how harmful was Mary's action as Very Much (6). Again, this is only a test for this story. Continue with the following questions. Mary sits quietly and continues eating his meal.

Questions battery

1. To what extent should Peter's action be punished?

Not at all Moderately Very much

0———-1———2———3———4———5——-—6

2. If you had witnessed Peter's action, how likely would you report it to competent authorities?

Very unlikely Not decided Very Likely

0———-1———2———3———4———5——-—6

3. How much harm was caused to Bilal?

Nothing at all Moderate Extreme

0———-1———2———3———4———5——-—6

EXPLORATORY STUDY: Sensitivity to verbal actions (Verbal-action Aversion Test)

How upset would it make you to curse angrily at an old woman as part of a movie script?

Not at all Moderately Extremely

0———-1———2———3———4———5——-—6

How upset would it make you to stab a fellow actor in the neck during a play using a stage knife with a retractable blade?

How anxious would it make you to give a speech in front of a large crowd?

How anxious would it make you to compete in a sports competition?

How happy would it make you to hear a colleague speaking well of you accidentally?

How angry would it make you to see your favourite sport-team lose a championship game?

How angry would it make you to see your best friend insulting an immigrant?

How upset would it make you to shoot a bullet at a consenting friend while he's behind a bulletproof glass?

How good would you feel after your boss congratulates you for doing a great job?

How embarrassed would it make you to make obscene gestures directed at your best friend behind their back?

How upset would it make you to see a friend yelling derogatory remarks at her mother on the phone while holding down the mute button?

How embarrassed would you feel if you realized you had toilet paper stuck to your shoe?

Supplementary Information: Appendix 2

Preliminary analysis: Assumptions tested for Multivariate Analysis of Variance.

We tested for multivariate outliers by computing Mahalanobis distance. 18 participants from 1309 were identified as multivariate outliers (p < .001) and removed according to the pre-reg protocol. Linearity assumptions were tested via scatterplots (see Supplementary Figure S1)

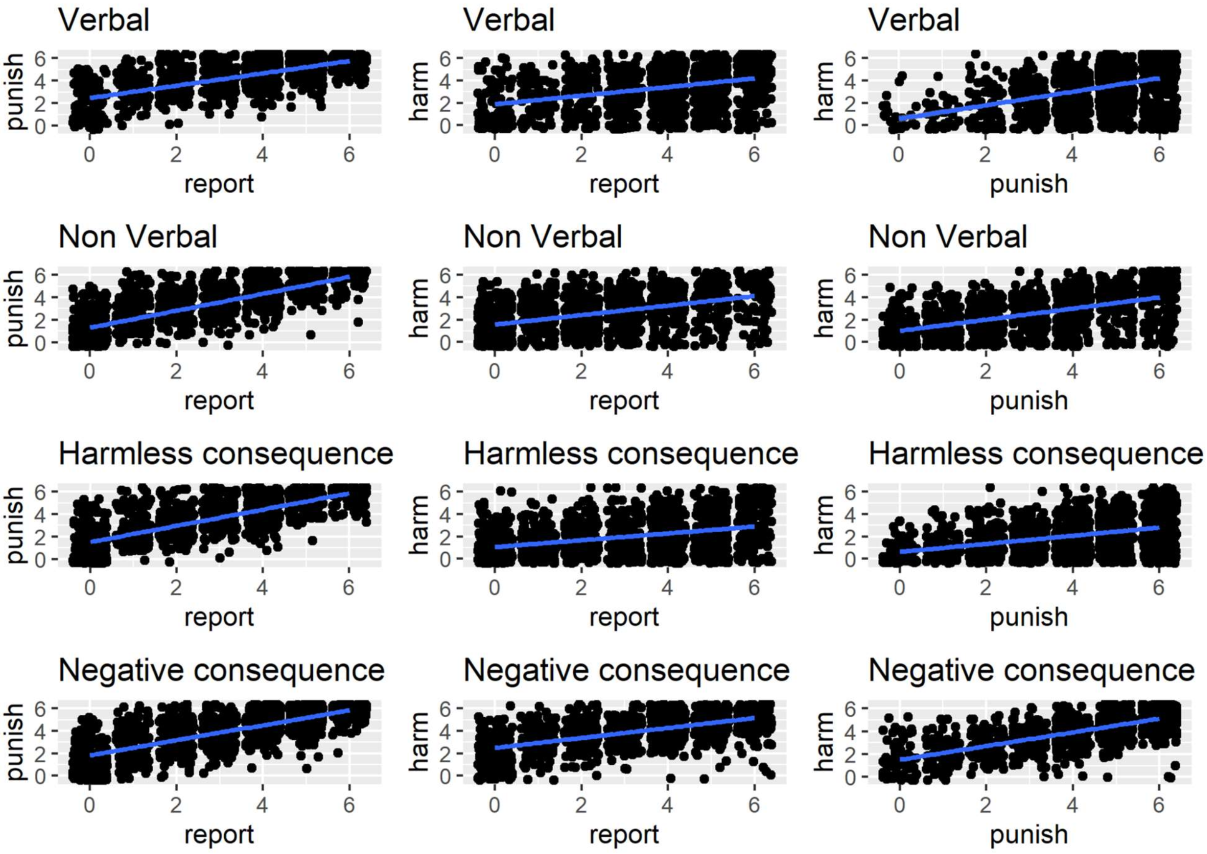

Supplementary Figure S1. *Scatterplots for each combination of dependent variables grouped by action type (verbal, nonverbal) and consequence_type (harmless consequence, negative consequence).*

The assumption of homogeneity of variance was assessed via Box M and Levene's test with multiple independent variables. Results for Box M show a p < .05 for all variables and as such violate the assumption of homogeneity of variance. Levene's tests were conducted and showed violations (p < .001). Mardia's skewness test was conducted and violations of this assumption were found with p < .001. To test for no multicollinearity, Spearman correlations were conducted for all dependent variables. No multicollinearity, rho > .90, was observed for all dependent variables (range rho = 0.46 - 0.77).

As seen by the results, the dependent variables are ordinal and the assumptions required for the MANOVA have been found to be violated; hence Johansen's general formulation of Welch-James's statistic with Approximate Degrees of Freedom (ADF) will be applied.

Assumption testing for Analysis of Variance.

The assumption of no outliers was assessed by using box m. No outliers were detected. Normality was assessed via histograms and qq-plots (see Supplementary Figures S2-7)

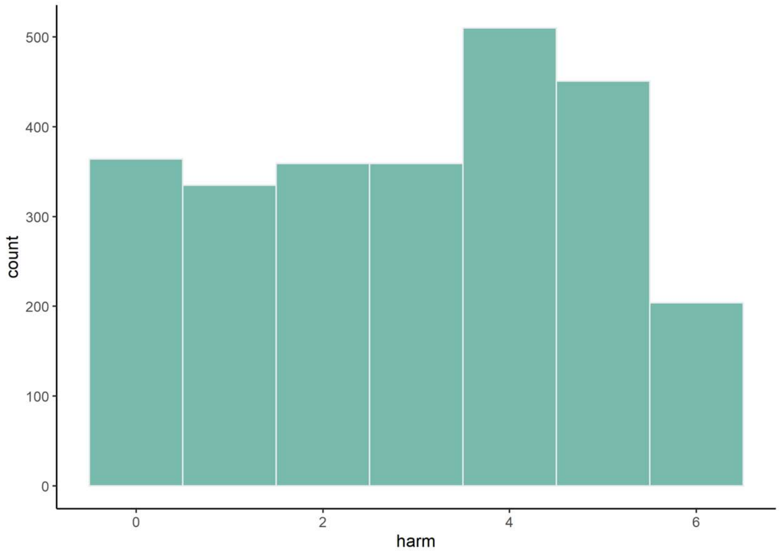

Supplementary Figure S2. *Histogram plot for level of harm*

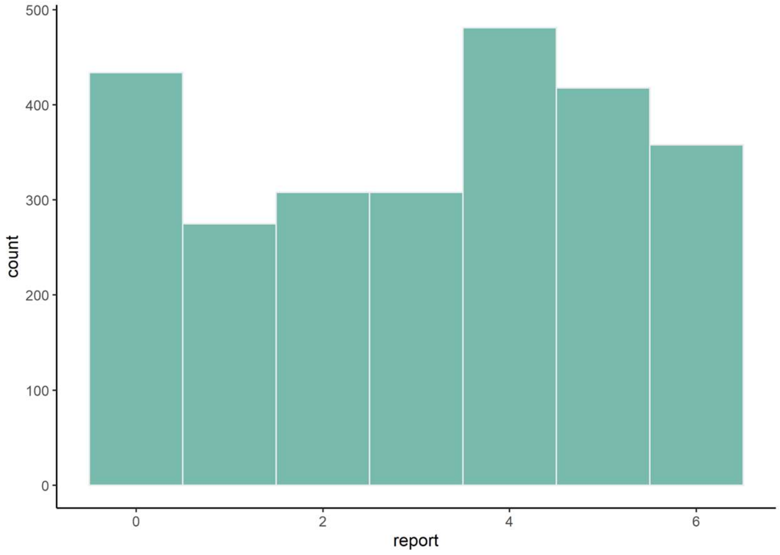

Supplementary Figure S3. *Histogram plot for likelihood to report*

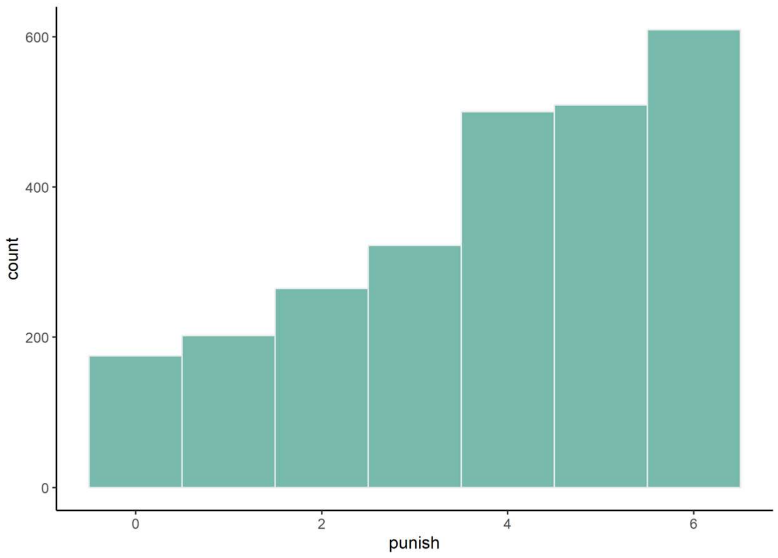

Supplementary Figure S4. *Histogram plot for appropriate punishment*

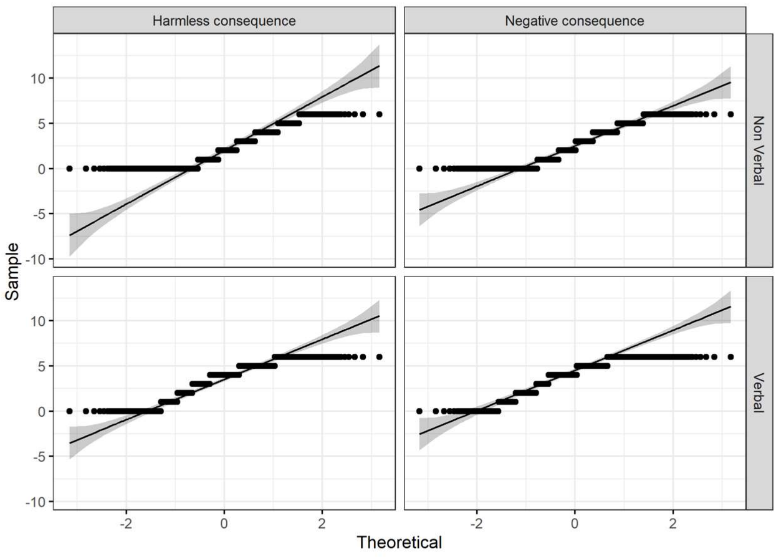

Supplementary Figure S5. *qq-plot for likelihood to report*

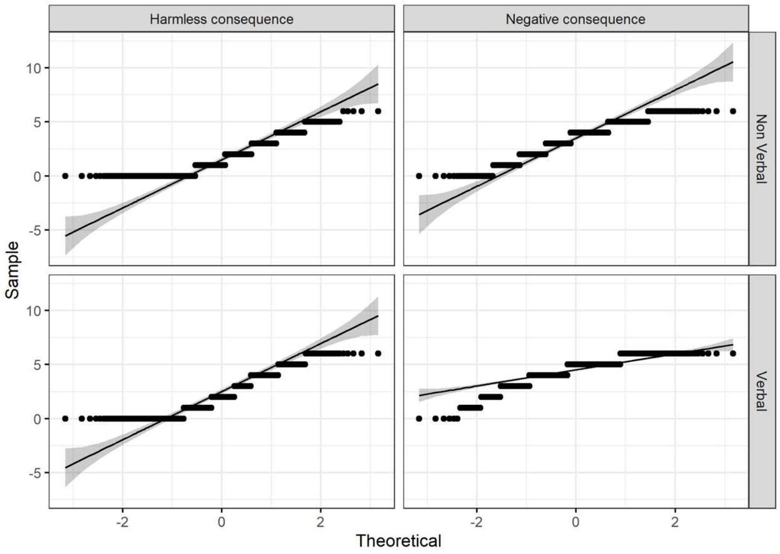

Supplementary Figure S6. *qq-plot for level of harm*

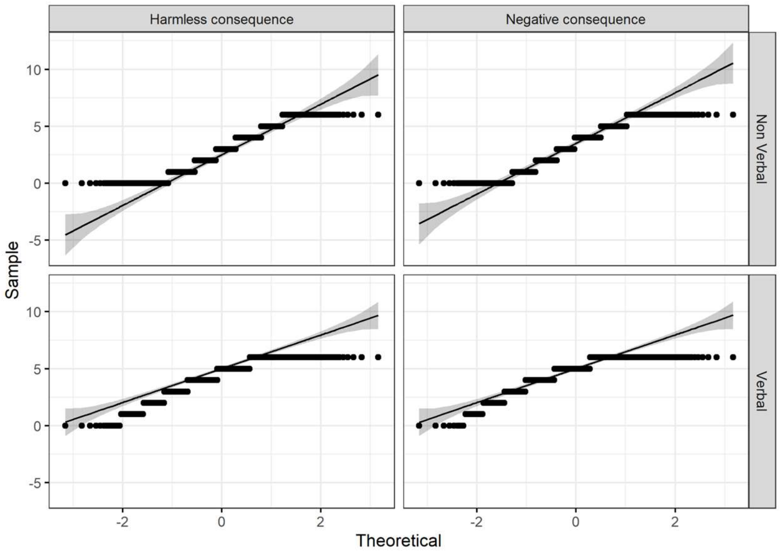

Supplementary Figure S7. *qq-plot for appropriate punishment*.

Homogeneity of variance is assessed via dot plots (see Figures B.7- 9.) and Levene's test. The Levene's test was significant ($p < 0.05$), so there was no homogeneity of variances.

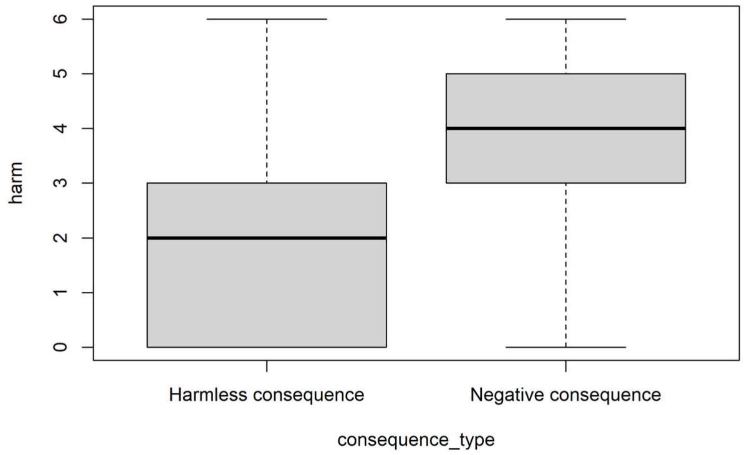

Supplementary Figure S8. *Dot plot of level of harm by consequence type*

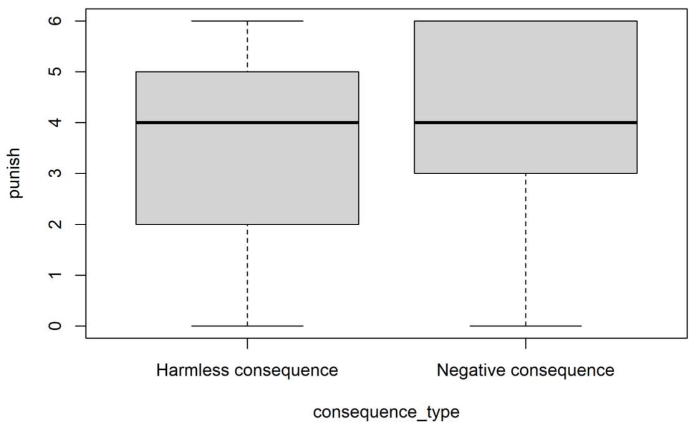

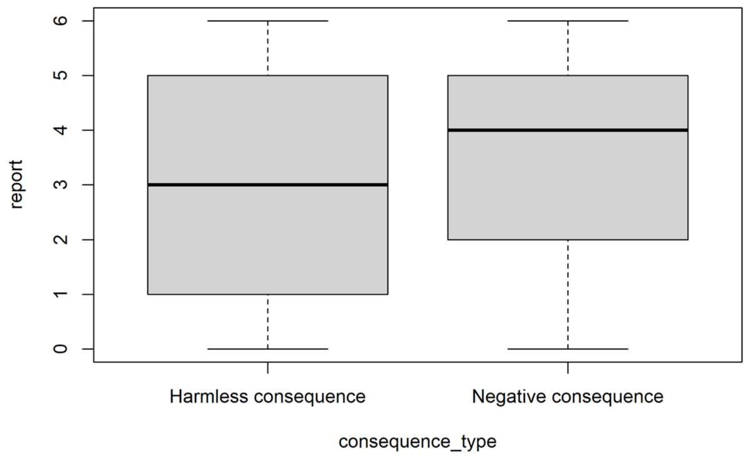

Supplementary Figure S9. *Dot plot of likelihood to report by consequence type*

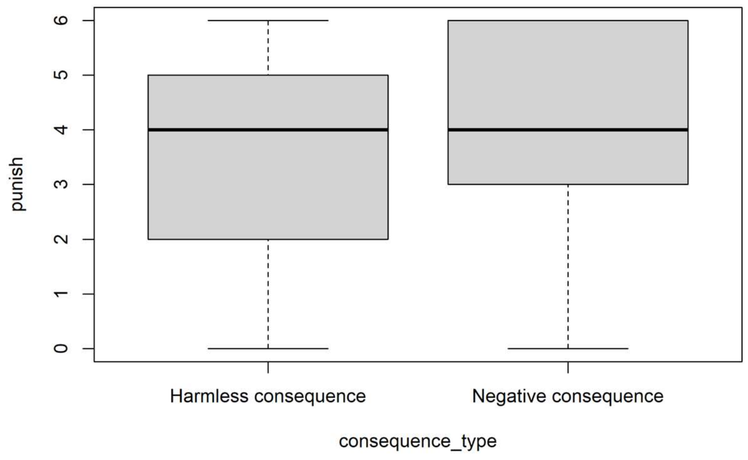

Supplementary Figure S10. *Dot plot of appropriate punishment by consequence type*

Finally, homogeneity of covariance was evaluated via Box's M test. Results were significant p< .05 and as such the assumption was violated.

Since the assumptions required for the parametric Analysis of Variance were violated, the non-parametric Welch-ADF test was conducted.

Supplementary Information: Appendix 3

Pre-registered Exploratory Analysis: Sensitivity to verbal actions (SVA)

Participants in our main study additionally answered the following questions:

1. How *upset* would it make you to curse angrily at an old woman as part of a movie script?
2. How *upset* would it make you to stab a fellow actor in the neck during a play using a stage knife with a retractable blade?
3. How *anxious* would it make you to give a speech in front of a large crowd?
4. How *anxious* would it make you to compete in a sports competition?
5. How *happy* would it make you to hear a colleague speaking well of you accidentally?
6. How *angry* would it make you to see your favourite sport-team lose a championship game?
7. How *angry* would it make you to see your best friend insulting an immigrant?
8. How *upset* would it make you to shoot a bullet at a consenting friend while he's behind a bulletproof glass?
9. How *good* would you feel after your boss congratulates you for doing a great job?
10. How *embarrassed* would it make you to make obscene gestures directed at your best friend behind their back?
11. How *upset* would it make you to see a friend yelling derogatory remarks at her mother on the phone while holding down the mute button?
12. How *embarrassed* would you feel if you realized you had toilet paper stuck to your shoe?

We used the following Likert scale for all the questions:

Not at all Moderately Extremely

0———-1———2———3———4———5—-—6

We operationalised the ***sensitivity to verbal actions (SVA)*** by calculating the mean scores of participants' ratings on how upset, anxious, happy, angry, good, embarrassed

disgusting or disturbing they reported to feel themselves in situations 1, 3, 5, 7, 9 and 11 we presented to them.

After collecting the data, we analysed whether the mean scores of SVA would correlate with/predict participants' mean ratings of verbal hate actions in the main experiment for the three DVs.

Correlation analysis

To test associations between participants' mean ratings of the dependent variables for verbal actions and SVA-mean scores, we conducted Spearman Correlations.

The Spearman's rank correlation rho between participants' mean ratings of the DV level of harm and the SVA-mean was positive, statistically significant, and medium (rho = 0.23, S = 2.86e+08, p < .001).

The Spearman's rank correlation rho between participants' mean ratings of the DV likelihood of denouncing and the SVA-mean was positive, statistically significant, and medium (rho = 0.23, S = 2.87e+08, p < .001).

The Spearman's rank correlation rho between participants' mean ratings of the DV deserved punishment and the SVA-mean was positive, statistically significant, and medium (rho = 0.27, S = 2.71e+08, p < .001)

Regression analysis

We then ran regression models to investigate further the relationships between the mean scores of SVA and the mean ratings of verbal hate actions in the main experiment for the three DVs.

We fitted a linear model (estimated using OLS) to predict harm ratings with SVA-mean (formula: harm ~ SVA-mean). The model explains a statistically significant and weak proportion of variance (R2 = 0.05, F(1, 1306) = 74.61, p < .001, adj. R2 = 0.05). The model's intercept, corresponding to SVA-mean = 0, is at 0.34 (95% CI [-0.17, 0.85], t(1306) = 1.30, p = 0.193). Within this model: The effect of the SVA-mean was statistically significant and positive (beta = 0.53, 95% CI [0.41, 0.66], t(1306) = 8.64, p < .001; Std. beta = 0.23, 95% CI [0.18, 0.29]). Standardised parameters were obtained by

fitting the model on a standardised version of the dataset. 95% Confidence Intervals (CIs) and p-values were computed using a Wald t-distribution approximation.

We fitted a linear model (estimated using OLS) to predict the likelihood of denouncing' ratings with SVA-mean (formula: report ~ SVA-mean). The model explains a statistically significant and weak proportion of variance ($R2 = 0.06$, $F(1, 1306) = 78.05$, $p < .001$, adj. $R2 = 0.06$). The model's intercept, corresponding to SVA-mean = 0, is at -0.19 (95% CI [-0.75, 0.38], $t(1306) = -0.65$, $p = 0.518$). Within this model: The effect of SVA-mean was statistically significant and positive (beta = 0.60, 95% CI [0.47, 0.73], $t(1306) = 8.83$, $p < .001$; Std. beta = 0.24, 95% CI [0.18, 0.29])

We fitted a linear model (estimated using OLS) to predict punishment ratings with SVA-mean (formula: punish ~ SVA-mean). The model explains a statistically significant and weak proportion of variance ($R2 = 0.08$, $F(1, 1306) = 107.22$, $p < .001$, adj. $R2 = 0.08$). The model's intercept, corresponding to SVA-mean = 0, is at 0.26 (95% CI [-0.29, 0.81], $t(1306) = 0.92$, $p = 0.355$). Within this model: The effect of the SVA-mean was statistically significant and positive (beta = 0.68, 95% CI [0.55, 0.81], $t(1306) = 10.35$, $p < .001$; Std. beta = 0.28, 95% CI [0.22, 0.33])

Supplementary Information: Appendix 4

Exploratory Analysis 2: Regression analysis to test potential interactions with demographics

To further analyse the data collected, we tested if demographic data collected show an interaction with action-type for the three DVs. We (re)coded verbal hate actions as 0 and nonverbal as 1.

For the dependent variable the **level of harm inflicted on the victim** we fitted a CLMM model. The model specification was as follows: **harm ~ action recoded*(Age + Gender + Social_pol_at + Edu + Discrimination + legal_sanction) + (1|ID)**. Results showed a significant main effect of action_recode1 (verbal coded as 0 and nonverbal coded as 1) (β = -0.007, p = .005), and of Gender Other (β = -2.951, p = .039), Social_pol_at: Very liberal (β = 0.012, p < .001), Edu: Master's Degree (β = 0.495, p <.001), as well as Discrimination: More than once ((β = 0.012, p < .001) and Discrimination: Never (β =1.086, p < .001). In addition, there was a significant interaction for action_recode1 and Age (β = -0.011, p < .001), for action_recode1 and Social_pol_at: Very liberal (β=0.251, p < .001), for action_recode1 and EduMaster's Degree (β =0.4762569, p <0.001), for action_recode1 and Discrimination Frequently (β = -0.9833831, p = .042), for action_recode1 and Discrimination More than once (β = -1.3236174, p < .001, as well as for action_recode1 and Discrimination Never (β = -1.043, p < .001).

term	estimate	std.error	statistic	p.value	conf.low	conf.high
0|1	-3.295181	0.0023767	-1386.478106	<0.001	-3.2998392	-3.2905228
1|2	-1.7499713	0.0022519	-777.1086343	<0.001	-1.7543849	-1.7455576
2|3	-0.5357112	0.0847341	-6.3222591	<0.001	-0.7017871	-0.3696353
3|4	0.5107739	0.1064874	4.7965653	<0.001	0.3020624	0.7194854
4|5	2.2059762	0.1330317	16.5823367	<0.001	1.9452389	2.4667135
5|6	4.726781	0.1842237	25.6578314	<0.001	4.3657091	5.0878528
action_recode1	-0.0069716	0.0024706	-2.8218045	0.005	-0.0118139	-0.0021293
Age	-0.0029166	0.0016024	-1.8201452	0.069	-0.0060572	0.000224
GenderMale	-0.2356328	0.2365951	-0.9959327	0.319	-0.6993506	0.2280851
GenderOther	-2.9506399	1.4268705	-2.0679101	0.039	-5.7472547	-0.1540251
GenderPrefer not to say	-3.3538566	1.8710506	-1.7924992	0.073	-7.0210484	0.3133351
Social_pol_atModerate	0.0139047	0.2450025	0.0567533	0.955	-0.4662914	0.4941008
Social_pol_atVery conservative	-0.2915687	0.8072307	-0.3611962	0.718	-1.8737118	1.2905745
Social_pol_atVery liberal	0.0122863	0.0024079	5.102571	<0.001	0.007567	0.0170057
Edu4-year College Degree	-0.0288827	0.2746293	-0.1051699	0.916	-0.5671463	0.5093808

EduDoctoral Degree	-0.7113891	0.8218723	-0.8655713	0.387	-2.3222292	0.899451
EduHigh School / GED	-0.0910601	0.3925481	-0.2319719	0.817	-0.8604404	0.6783201
EduLess than High School	-1.4463609	1.9709243	-0.733849	0.463	-5.3093015	2.4165798
EduMaster’s Degree	0.4946808	0.0024078	205.4497444	<0.001	0.4899616	0.4994
EduProfessional Degree (JD, MD)	0.9036435	0.8578443	1.0533887	0.292	-0.7777004	2.5849875
EduSome College	0.4155358	0.3564437	1.1657824	0.244	-0.283081	1.1141525
DiscriminationFrequently	0.9355556	0.5526214	1.6929413	0.09	-0.1475624	2.0186737
DiscriminationMore than once	1.0236041	0.2583514	3.9620613	<0.001	0.5172447	1.5299636
DiscriminationNever	1.0857483	0.0024077	450.9485866	<0.001	1.0810293	1.0904673
DiscriminationOnly once	0.3003517	0.4795562	0.6263118	0.531	-0.6395611	1.2402645
legal_sanctionSurely yes	0.2253519	0.2424967	0.9292991	0.353	-0.2499328	0.7006366
action_recode1:Age	-0.010594	0.0014342	-7.3865628	<0.001	-0.013405	-0.007783
action_recode1:GenderMale	-0.3265413	0.202634	-1.6114838	0.107	-0.7236966	0.0706139
action_recode1:GenderOther	1.0854986	1.1570139	0.9381898	0.348	-1.1822069	3.3532042
action_recode1:GenderPrefer not to say	-0.9839807	1.8585263	-0.5294414	0.596	-4.6266253	2.6586639
action_recode1:Social_pol_atModerate	-0.1786634	0.2102016	-0.8499619	0.395	-0.5906509	0.2333242
action_recode1:Social_pol_atVery conservative	-0.0819349	0.7154177	-0.1145274	0.909	-1.4841278	1.3202579
action_recode1:Social_pol_atVery liberal	0.2508663	0.0024706	101.5395287	<0.001	0.2460239	0.2557086
action_recode1:Edu4-year College Degree	0.4568969	0.2337176	1.9549103	0.051	-0.0011811	0.9149749
action_recode1:EduDoctoral Degree	1.2507887	0.6609661	1.8923644	0.058	-0.044681	2.5462584
action_recode1:EduHigh School / GED	0.1841568	0.3394444	0.5425242	0.587	-0.4811421	0.8494557
action_recode1:EduLess than High School	-2.6349599	2.1573825	-1.2213689	0.222	-6.863352	1.5934322
action_recode1:EduMaster’s Degree	0.4762569	0.0024706	192.7666185	<0.001	0.4714146	0.4810993
action_recode1:EduProfessional Degree (JD, MD)	-0.9286142	0.7527652	-1.2336041	0.217	-2.4040069	0.5467785
action_recode1:EduSome College	0.0557152	0.3068773	0.1815553	0.856	-0.5457533	0.6571837
action_recode1:DiscriminationFrequently	-0.9833831	0.4830433	-2.0358074	0.042	-1.9301305	-0.0366357
action_recode1:DiscriminationMore than once	-1.3236174	0.2197128	-6.0243079	<0.001	-1.7542465	-0.8929883

action_recode1:DiscriminationNever	-1.042718	0.0024706	-422.0477708	<0.001	-1.0475603	-1.0378757
action_recode1:DiscriminationOnly once	-0.5249594	0.4193037	-1.2519789	0.211	-1.3467795	0.2968608
action_recode1:legal_sanctionSurely yes	-0.2059287	0.2074211	-0.9928048	0.321	-0.6124666	0.2006092

For the dependent variable **appropriate punishment** we fitted a CLMM model. The model specification was as follows: **punish ~ action recoded*(Age + Gender + Social_pol_at + Edu + Discrimination + legal_sanction) + (1|ID)**. We found a significant main effect of Age (β =-0.023 , p = .005), Discrimination: Frequently (β = 3.493, p = 0.007), Discrimination: More than once (β =2.955, p = .015), Discrimination: Never (β = 3.163, p = .010), Discrimination: Only once (β =2.955, p = .021) and Legal_sanction: Surely yes (β =0.731, p < .001). There was also significant interaction between action_recode1 and legal_sanction: Surely yes (β =-0.579, p = .009)

term	estimate	std.error	statistic	p.value	conf.low	conf.high
0|1	-3.4326124	1.3225749	-2.5954011	0.009	-6.0248116	-0.8404132
1|2	-2.3681284	1.3180914	-1.7966344	0.072	-4.9515401	0.2152833
2|3	-1.2704232	1.3146151	-0.9663842	0.334	-3.8470214	1.306175
3|4	-0.2894649	1.3131887	-0.220429	0.826	-2.8632675	2.2843376
4|5	0.9649758	1.3137989	0.7344928	0.463	-1.6100228	3.5399744
5|6	2.4589471	1.3172329	1.866752	0.062	-0.1227818	5.0406761
action_recode1	-0.0590114	1.387284	-0.0425373	0.966	-2.778038	2.6600153
Age	-0.0238383	0.0085752	-2.7799146	0.005	-0.0406454	-0.0070312
GenderMale	-0.0287963	0.2100676	-0.1370812	0.891	-0.4405212	0.3829285
GenderOther	-1.7036347	1.125051	-1.5142733	0.13	-3.9086941	0.5014247
GenderPrefer not to say	0.1323414	1.4974179	0.0883798	0.93	-2.8025437	3.0672265
Social_pol_atModerate	-0.1939197	0.2266821	-0.85547	0.392	-0.6382085	0.250369
Social_pol_atVery conservative	-0.8679348	0.6484039	-1.3385712	0.181	-2.1387832	0.4029135
Social_pol_atVery liberal	0.1641331	0.2990737	0.5488048	0.583	-0.4220406	0.7503068
Edu4-year College Degree	-0.6165268	0.4058118	-1.5192433	0.129	-1.4119033	0.1788496
EduDoctoral Degree	-1.2351477	0.7229736	-1.708427	0.088	-2.65215	0.1818546
EduHigh School / GED	-0.0478428	0.4613292	-0.1037065	0.917	-0.9520315	0.8563458

EduLess than High School	-0.7343856	1.4575583	-0.5038465	0.614	-3.5911474	2.1223761
EduMaster's Degree	-0.3958247	0.4532131	-0.8733743	0.382	-1.284106	0.4924567
EduProfessional Degree (JD, MD)	-0.7342316	0.7763547	-0.9457425	0.344	-2.2558588	0.7873956
EduSome College	-0.1878741	0.4401003	-0.4268893	0.669	-1.0504549	0.6747067
DiscriminationFrequently	3.4925026	1.287779	2.7120356	0.007	0.9685021	6.016503
DiscriminationMore than once	2.9548233	1.2190827	2.4238088	0.015	0.5654651	5.3441814
DiscriminationNever	3.1631049	1.2205587	2.5915221	0.01	0.7708538	5.5553559
DiscriminationOnly once	2.9056353	1.2596454	2.3067089	0.021	0.4367757	5.3744949
legal_sanctionSurely yes	0.7310426	0.209014	3.4975766	<0.001	0.3213826	1.1407026
action_recode1:Age	-0.0043194	0.0091756	-0.470756	0.638	-0.0223032	0.0136643
action_recode1:GenderMale	-0.3386928	0.2266079	-1.4946206	0.135	-0.7828361	0.1054505
action_recode1:GenderOther	-0.8241553	1.1783652	-0.6994057	0.484	-3.1337087	1.4853981
action_recode1:GenderPrefer not to say	0.0625891	1.6298125	0.0384026	0.969	-3.1317847	3.2569628
action_recode1:Social_pol_atModerate	0.328701	0.2433643	1.3506541	0.177	-0.1482843	0.8056864
action_recode1:Social_pol_atVery conservative	-0.0280045	0.7191212	-0.0389427	0.969	-1.437456	1.3814471
action_recode1:Social_pol_atVery liberal	0.3013427	0.3222266	0.9351888	0.35	-0.3302098	0.9328953
action_recode1:Edu4-year College Degree	0.6430689	0.4329682	1.4852567	0.137	-0.2055332	1.4916709
action_recode1:EduDoctoral Degree	1.1594003	0.7593567	1.5268191	0.127	-0.3289115	2.6477121
action_recode1:EduHigh School / GED	0.4644105	0.4961787	0.9359742	0.349	-0.508082	1.4369029
action_recode1:EduLess than High School	-1.0860831	1.4852127	-0.7312643	0.465	-3.9970465	1.8248803
action_recode1:EduMaster's Degree	0.2368252	0.4840782	0.4892292	0.625	-0.7119507	1.1856011
action_recode1:EduProfessional Degree (JD, MD)	-0.7476246	0.8414762	-0.8884679	0.374	-2.3968876	0.9016384
action_recode1:EduSome College	0.6749977	0.4707044	1.434016	0.152	-0.247566	1.5975614
action_recode1:DiscriminationFrequently	-1.665322	1.3560811	-1.2280401	0.219	-4.3231922	0.9925482
action_recode1:DiscriminationMore than once	-2.4291964	1.2830645	-1.8932769	0.058	-4.9439566	0.0855638
action_recode1:DiscriminationNever	-2.3451826	1.2846443	-1.8255501	0.068	-4.8630393	0.172674
action_recode1:DiscriminationOnly once	-2.2919164	1.3297862	-1.7235225	0.085	-4.8982494	0.3144166

action_recode1:legal_sanctionSurely yes	-0.5798991	0.2233201	-2.5967166	0.009	-1.0175986	-0.1421997

For the dependent variable the **likelihood of denouncing perpetrators to competent authorities** we fitted a CLMM model. The model specification was as follows: **report ~ action recoded*(Age + Gender + Social_pol_at + Edu + Discrimination + legal_sanction) + (1|ID)**. There was a significant main effect of Social_pol_at: Very conservative (β = -1.554 , p = .018), of Social_pol_atVery liberal (β =0.636, p = .029), Discrimination:Frequently (β =2.880 , p = .025), and legal_sanctionSurely yes (β =0.644, p = .001). As well as a significant interaction between action_recode1:legal_sanctionSurely yes (β =-0.535 , p = .014)

term	estimate	std.error	statistic	p.value	conf.low	conf.high
0|1	-1.14871	1.3119587	-0.8755687	0.381	-3.7201018	1.4226819
1|2	-0.2625637	1.3107589	-0.2003143	0.841	-2.831604	2.3064766
2|3	0.6048652	1.3107041	0.4614811	0.644	-1.9640677	3.1737981
3|4	1.3999824	1.3113221	1.0676114	0.286	-1.1701617	3.9701265
4|5	2.8049481	1.3150088	2.133026	0.033	0.2275781	5.382318
5|6	4.2025816	1.3217975	3.1794445	0.001	1.6119062	6.793257
action_recode1	-0.4025529	1.3603251	-0.295924	0.767	-3.0687411	2.2636353
Age	-0.0066921	0.0082725	-0.8089536	0.419	-0.0229059	0.0095217
GenderMale	0.0474505	0.2029472	0.2338073	0.815	-0.3503187	0.4452197
GenderOther	-1.6318063	1.1391256	-1.4325078	0.152	-3.8644514	0.6008388
GenderPrefer not to say	0.0377415	1.4463977	0.0260934	0.979	-2.7971459	2.8726289
Social_pol_atModerate	-0.3676619	0.2195182	-1.6748582	0.094	-0.7979097	0.0625859
Social_pol_atVery conservative	-1.5549693	0.6571675	-2.366169	0.018	-2.8429939	-0.2669447
Social_pol_atVery liberal	0.636319	0.2915679	2.1824041	0.029	0.0648564	1.2077817
Edu4-year College Degree	0.2323586	0.3859635	0.6020222	0.547	-0.524116	0.9888332
EduDoctoral Degree	-0.1646026	0.7151421	-0.2301676	0.818	-1.5662554	1.2370502
EduHigh School / GED	0.3898264	0.4388161	0.8883592	0.374	-0.4702375	1.2498902
EduLess than High School	-2.2204985	1.3841178	-1.6042698	0.109	-4.9333196	0.4923227
EduMaster’s Degree	0.6238229	0.4345489	1.4355644	0.151	-0.2278773	1.475523
EduProfessional Degree (JD, MD)	0.4472451	0.7492366	0.5969343	0.551	-1.0212318	1.9157219
EduSome College	0.5022582	0.4219276	1.1903895	0.234	-0.3247048	1.3292212
DiscriminationFrequently	2.8805526	1.2854386	2.2409103	0.025	0.3611393	5.3999659

DiscriminationMore than once	2.3351104	1.2222009	1.9105782	0.056	-0.0603594	4.7305802
DiscriminationNever	2.362143	1.2224207	1.9323487	0.053	-0.0337574	4.7580435
DiscriminationOnly once	1.8586747	1.2560999	1.4797188	0.139	-0.6032359	4.3205854
legal_sanctionSurely yes	0.6444034	0.2015922	3.1965689	0.001	0.2492899	1.0395169
action_recode1:Age	-0.0013403	0.0090062	-0.1488177	0.882	-0.0189921	0.0163115
action_recode1:GenderMale	-0.2850092	0.2210961	-1.2890736	0.197	-0.7183496	0.1483312
action_recode1:GenderOther	0.1296991	1.1890657	0.1090764	0.913	-2.2008268	2.4602249
action_recode1:GenderPrefer not to say	1.1298091	1.656617	0.6819978	0.495	-2.1171005	4.3767187
action_recode1:Social_pol_atModerate	0.2403004	0.2381311	1.0091097	0.313	-0.226428	0.7070289
action_recode1:Social_pol_atVery conservative	0.4247233	0.7326532	0.5797058	0.562	-1.0112505	1.8606971
action_recode1:Social_pol_atVery liberal	0.0319503	0.3174739	0.1006392	0.92	-0.5902871	0.6541877
action_recode1:Edu4-year College Degree	0.1400314	0.4239335	0.3303145	0.741	-0.690863	0.9709258
action_recode1:EduDoctoral Degree	0.0415493	0.7801683	0.0532568	0.958	-1.4875525	1.570651
action_recode1:EduHigh School / GED	0.1467376	0.4824894	0.304126	0.761	-0.7989242	1.0923993
action_recode1:EduLess than High School	0.6825656	1.4733645	0.46327	0.643	-2.2051757	3.570307
action_recode1:EduMaster’s Degree	-0.2589107	0.4761242	-0.5437882	0.587	-1.192097	0.6742756
action_recode1:EduProfessional Degree (JD, MD)	-1.0705917	0.8242587	-1.2988539	0.194	-2.6861091	0.5449257
action_recode1:EduSome College	0.2694311	0.4628691	0.5820891	0.561	-0.6377758	1.1766379
action_recode1:DiscriminationFrequently	-0.7702441	1.3259539	-0.5808981	0.561	-3.3690659	1.8285777
action_recode1:DiscriminationMore than once	-1.7631153	1.2552335	-1.4046114	0.16	-4.2233277	0.6970972
action_recode1:DiscriminationNever	-1.4298245	1.2555485	-1.1388047	0.255	-3.8906545	1.0310054
action_recode1:DiscriminationOnly once	-1.006824	1.2951958	-0.7773527	0.437	-3.5453612	1.5317132
action_recode1:legal_sanctionSurely yes	-0.5358009	0.2181288	-2.4563516	0.014	-0.9633255	-0.1082764
